# Characteristics, clinical evidence and implementation effects of conditional approvals for drugs in China, a pooled analysis from 2020 to 2023

**DOI:** 10.3389/fphar.2025.1501525

**Published:** 2025-04-25

**Authors:** Li Yang, Shuoxin Fan, Jie Zhang, Geyun Huang, Yaoyang Tang, Lijia Xue

**Affiliations:** School of Business Administration, Shenyang Pharmaceutical University, Shenyang, China

**Keywords:** conditional approvals, characteristics, clinical evidence, implementation effects, China

## Abstract

In late 2019, the conditional approval process for drugs in China transitioned from a pilot project to a formal program. Our study comprehensively analyzed 103 conditional approvals (CAs) authorized by the National Medical Products Administration (NMPA) from 2020 to 2023, specifically focusing on their characteristics, clinical evidence, and implementation effects. It also explored the challenges faced by the CA program in China. The primary findings indicated that nearly 90% of China’s CAs were granted for antineoplastics agents, and there were no reported cases of CAs withdrawn by NMPA from the market. Notably, a substantial disparity existed in the pivotal premarketing and completed/ongoing postmarketing clinical trial features and endpoints. Additionally, CAs which initiated confirmation clinical trials before the CA application submission were more likely to obtain regular approval. The efficacy evidence of CAs supported by single-arm trials demonstrated statistically significant variances in indication and drug types. However, no statistical distinctions were observed in the efficacy evidence of CAs supported by randomized controlled trials (RCTs). CAs have been shown to decrease the development time, review time, and drug lag period, notably compared to non-CAs, and differences exist in the development time, review time, and drug lag period among CAs. Furthermore, numerous unmet clinical needs and the public health emergency of COVID-19 have been partially addressed through CAs. However, the approval procedures, clinical evidence evaluation systems, pharmacovigilance, and requirements for confirmatory trials within China’s CA framework still require further enhancement.

## 1 Introduction

To address the issue of patient access to urgently needed medicines and expedite the review and approval of drugs, China initiated a reform of its regulatory system in 2015 (State Council of the People’s Republic of China, 2015). A pilot program for conditional approval (CA) was launched in 2017, focusing on drugs crucial for public health or treating diseases that seriously endanger life and have no effective treatment methods. If early and mid-term clinical trial indicators show efficacy and can predict clinical value, they can be conditionally approved for marketing (State Council of the People’s Republic of China, 2017). In 2019, the CA program was officially integrated into the “Drug Administration Law” and “Vaccine Administration Law “of China (National Medical Products Administration, 2024a; National Medical Products Administration, 2019). Subsequently, the National Medical Products Administration (NMPA) issued the “Drug Registration Regulation” in 2020 to specify the applicable circumstances, confirmatory trial submission, and risk management requirements for CAs (National Medical Products Administration, 2020a). Furthermore, a series of regulatory documents from the NMPA and technical guidelines from the Center for Drug Evaluation (CDE) within the NMPA clarified the process and specific technical criteria for the evaluation and authorization of CAs. Currently, the CA process for drugs in China is applicable in situations where the drug is intended for life-threatening diseases with no existing curative treatments or in cases where the drug is urgently required for public health reasons. The CA program is contingent upon the drug’s demonstrated effectiveness in clinical trials and anticipated clinical benefits. Furthermore, CA holders must conduct postmarketing clinical trials within a specified timeframe. After completing these trials, the NMPA will assess the results and determine whether to grant regular approvals for the products or remove them from the market (Center For Drug Evaluation and NMPA, 2020; National Medical Products Administration, 2020d). Currently, limited research exists about the CAs of China. This study conducted a comprehensive analysis of the CAs authorized in China between 2020 (the year making the formal inception of the CA program in China) and 2023, evaluated the characteristics, clinical evidence, and implementation effects of the CAs, and discussed the potential challenges within the CA framework.

## 2 Methods

### 2.1 Study design and sample

This research examined the conditional approvals of drugs by the NMPA of China between 2020 and 2023. In this study, the drug approved in a drug evaluation report and its approved specific indication were combined as a single entity for research, called an “approved item”, resulting in 103 approvals.

### 2.2 Data source and extraction

This study retrieved the list of CAs from the China Drug Review Annual Reports published by the CDE within NMPA from 2020 to 2023. By searching the official database of the CDE as well as the business database called YAOZHI China, we collected detailed data on the essential characteristics of the approvals, including the date of investigational new drug (IND) approval, new drug application (NDA) submission and NDA approval, the type of product (chemical drug, therapeutic biological product, or traditional Chinese medicine), drug registration category, therapeutic area to which the indication belongs, and the manufacturing source of the drug (National Medical Products Administration, 2024b; Yaozhi, 2024). The therapeutic area was categorized according to the World Health Organization Anatomical Therapeutic Chemical Classification System (ATC) (WHO Collaborating Centre for Drug Statistics Methodology, 2024), and the manufacturing source was classified as domestic or imported depending on the manufacturer’s location within or outside of mainland China.

This study collected the pivotal premarketing clinical trial evidence and postmarketing obligations for CAs from the drug evaluation reports released by the CDE (National Medical Products Administration, 2024b). Pivotal premarketing clinical trials were the primary clinical trials supported the conditional approvals and were labeled in the assessment section of CDE evaluation reports as “pivotal trial” or “primary evidence”. If one CA referred to more than one pivotal premarketing clinical trials, they would be all included. Additionally, this study also obtained some supplementary data by searching for Clinical Trials. gov and Chinadrugtrials. org.cn, including the primary efficacy endpoints, effect values, and design types of pivotal premarketing trials and postmarketing trials, as randomized controlled trials (RCTs) or single-arm trials, and the stage of clinical trials (phase Ⅰ, Ⅱ, Ⅲ, *etc.*, mentioned in the evaluation reports) supporting the approval of the products. We divided the postmarketing clinical trials into two categories: the first pertained to CAs that were still under the conditional status, referred to as ongoing postmarketing clinical trals; the second pertained to CAs that had completed the conversion to regular approvals, referred to as completed postmarketing clinical trials. As the CDE did not disclose the evaluation reports for COVID-19 management drugs, the statistics mentioned above did not include data about these drugs.

### 2.3 Statistics

Categorical variables were characterized by frequency and percentage. The chi-square or Fisher’s exact test was employed to compare the categorical information. The medians and their interquartile range (IQR) were represented as continuous variables. We used the Mann-Whitney U or the Kruskal–Wallis test to assess the disparity in numerical data.

Furthermore, this paper analyzed the efficacy and safety evidence of CAs by meta-analysis. A subgroup meta-analysis was conducted on the response rate (RR) outcomes and the duration of response (DOR) reported in single-arm trials and RCTs, as in the hazard ratio (HR) for the progression-free survival (PFS) and overall survival (OS) outcomes reported in RCTs. A meta-analysis was conducted on the safety outcomes, including grade 3 or higher AEs (including grade 3) (Grade ≥3 AEs) and serious adverse events (SAEs) from single-arm trials and RCTs in the CAs. The I^2^ statistic was used to assess the statistical heterogeneity. Usually, I^2^ ≥ 50% indicates significant heterogeneity, and the experiment should adopt the random-effects model; otherwise, a fixed-effects model would be performed (Sedgwick, 2015). P values were calculated based on Cochran’s Q test for subgroup differences.

Statistical analysis and plotting were performed using SPSS 27.0, R version 4.3.2 (R package meta, version 7.0–0), and GraphPad Prism 8.0. P-value <0.05 was considered a statistical significance; The term “95% CI” denoted a 95% confidence interval.

## 3 Results

### 3.1 Characteristics

#### 3.1.1 Coverage of conditional approvals

From 1 January 2020, to 31 December 2023, the NMPA granted CAs to 138 applications covering 103 approvals for 86 products. As of 31 January 2025, 25 CAs have completed postmarketing requirements and converted to regular approvals (RAs). Additionally, mobocertinib’s confirmatory trial failed to meet its primary endpoint, leading to the sponsor’s voluntary market withdrawal (Supplementary Table S2, see Supplementary Material).

**FIGURE 1:**
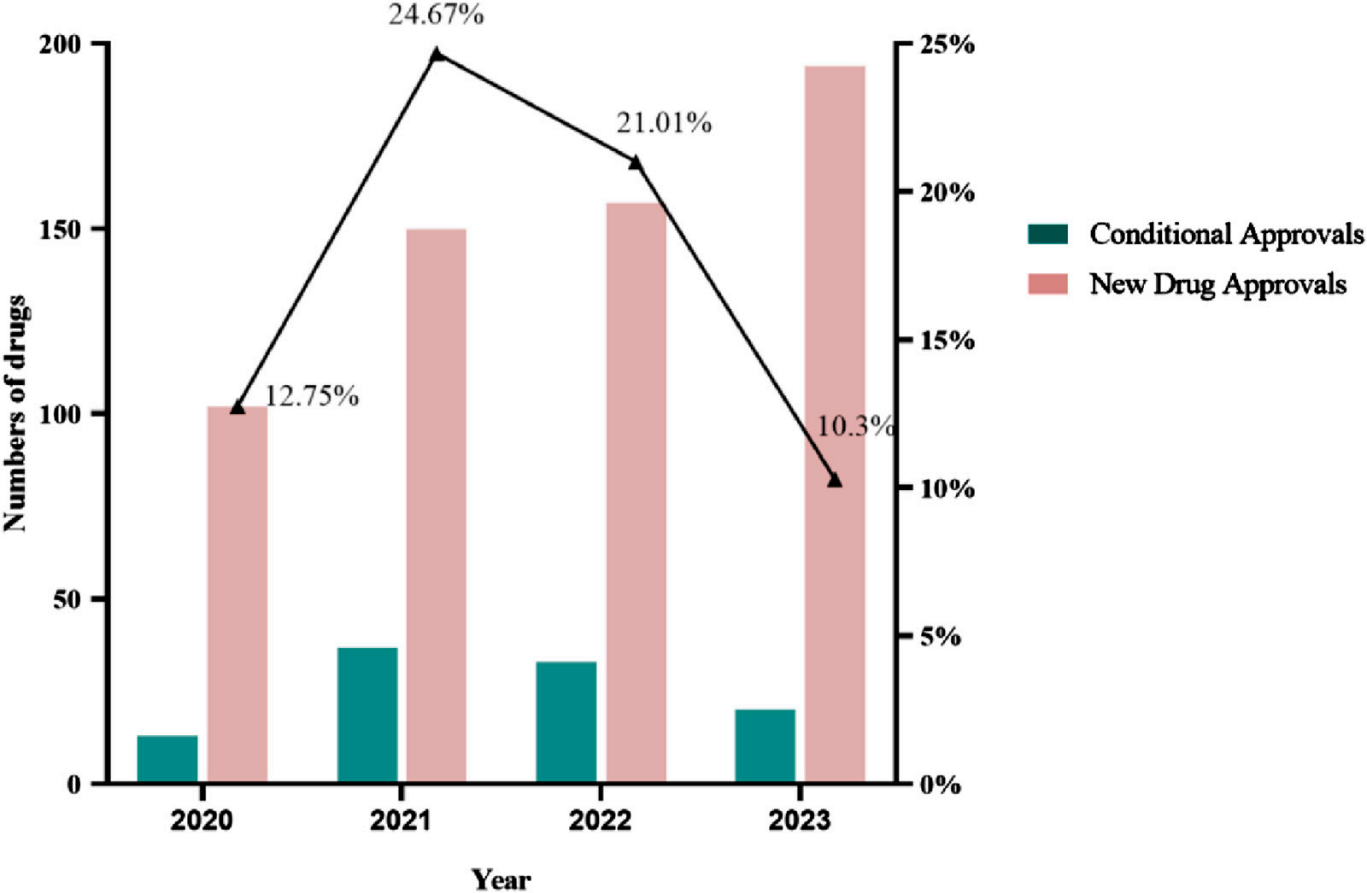
New drug approvals in China from 2020 to 2023.

The annual number of CAs in China increased from 2020 to 2021. In 2021, 24.67% of new drug approvals were granted with conditions, amounting to 37 approvals. From 2021 to 2023, the annual number of CAs decreased yearly. This change shows that there is a policy-tightening trend for the approval of CA-purpose drugs in China (Figure 1). In August 2023, CDE published the exposure draft of “Procedure on Review and Approval of the Market Application for Conditional Approval Drug” with a series of closed-door policies for CA in China (National Medical Products Administration, 2023a). The policies entail the disapproval of clinical trial applications for drugs intended for CAs if similar drugs have already received CAs and the disapproval of marketing applications for drugs intended for CAs if similar drugs have converted to regular approvals in China.

Out of the 103 approvals, 56 (54.4%) were for chemical drugs, 45 (43.7%) were for therapeutic biological products, and 2 (1.9%) were for traditional Chinese medicines (TCMs) (Table 1). Approvals for chemical drugs were classified 1, 2.2, 2.4, and 5.1, representing 22.3%, 1.0%, 4.9%, and 26.2% of the total, respectively (National Medical Products Administration, 2020b). Approvals for therapeutic biological products had the highest representation in class 1, followed by classes 2.2, 3.1, and 3.2. Among approvals for TCMs, 1 was classified as 1.1 and 1 as 1.2. The Drug Registration Regulation 2020 defined the registration categories. The precise definitions of registration classifications are available in Supplementary Table S3 (see Supplementary Material).

**TABLE 1:** Characteristics of CAs granted by the NMPA (2020–2023).

Characteristics	No. (%)
Drug type	
Chemical drugs Therapeutic biological products	56 (54.4) 45 (43.7)
Traditional Chinese medicines	2 (1.9)
Registration category	
Chemical drugs	
Class 1	23 (22.3)
Class 2.2	1 (1.0)
Class 2.4	5 (4.9)
Class 5.1	27 (26.2)
Therapeutic biologic products	
Class 1	22 (21.4)
Class 2.2	7 (6.8)
Class 3.1	15 (14.6)
Class 3.2	1 (1.0)
Traditional Chinese medicines	
Class 1.1	1 (1.0)
Class 1.2	1 (1.0)
Origin	
Domestic	61 (59.2)
Imported	42 (40.8)
Therapeutic area	
Antineoplastics agents	88 (85.4)
Immunomodulating agents	1 (1.0)
Anti-infective for systemic use	9 (8.7)
Blood and blood-forming organs	2 (1.9)
Musculoskeletal system	2 (1.9)
Dermatologicals	1 (1.0)
Expedited program	
CA	9 (8.7)
BTD + CA	1 (1.0)
PR + CA	71 (68.9)
BTD + PR + CA	14 (13.6)
CA + SRA	8 (7.8)

A total of 42 CAs were permitted for domestic sale despite being manufactured abroad, which were defined as imported. Out of the 103 CAs granted, 88 were for antineoplastics indications, while the remaining approvals encompassed a variety of conditions such as anti-infective, musculoskeletal, and hematopoietic system disorders. Specifically, 8 out of the 9 systemic anti-infective drugs are designed to target COVID-19 infection. In addition to the CA program, 3 other programs exist in China to accelerate drug development or review and enhance drug access: the Breakthrough Therapy Designation Program (BTD), the Priority Review Designation Program (PR), and the Special Review and Approval Procedure Program (SRA). These programs are not mutually exclusive, as drugs granted to CAs may also be eligible to apply for other programs. The introduction and comparison of the 4 expedited programs can be seen in Supplementary Table S1 (see Supplementary Material) (Li et al., 2021). According to the statistics, 94 (91.3%) CAs utilized more than 1 program. In total, 9 (8.7%) approvals were conditionally approved alone, while 1 (1.0%) approval received both CA and BTD. The predominant trend observed was 71 (68.9%) approvals obtained by CA and PR. Additionally, 14 (13.6%) approvals were concurrently granted CA, BTD, and PR. The 8 anti-COVID-19 drugs were simultaneously acquired through CA and SRA to expedite their market availability.

#### 3.1.2 Pivotal premarketing and completed/ongoing postmarketing clinical trial features and end points

Our analysis identified 117 pivotal premarketing clinical trials, and 84 postmarketing clinical trials (8 approvals for COVID-19 management drugs, along with 10 additional approvals lacking publicly accessible evaluation reports, as well as 1 withdrawn approval, were excluded from the analysis). Among the postmarketing clinical trials, 59 (70.2%) were for CAs which still under conditionl status, while 25 (29.8%) for CAs which had been under regular status, as shown in Table 2.

**TABLE 2:** Pivotal premarketing and completed/ongoing postmarketing clinical trial features and end points for CAs by NMPA, 2020–2023.

	Study type and status, NO. (%)			
Clinical trial characteristics	Pivotal premarketing clinical trials (n = 117)	Completed postmarketing clinical trials (n = 25)	Ongoing postmarketing clinical trials (n = 59)	P
Enrollment, median (IQR) No. of patients	107 (61–221)	289 (114–455)	161 (65–332)	<0.001^*^
Type of clinical trial				
Single-arm trials	91 (77.8)	6 (24)	23 (39)	<0.001
Randomized controlled trials	26 (22.2)	19 (76)	36 (61)	
Stage of clinical trial				
Phase Ⅰ	6 (5.1)	0 (0)	0 (0)	<0.001
Phase Ⅰ/Ⅱ	25 (21.4)	2 (8)	1 (1.7)	
Phase Ⅱ	60 (51.3)	3 (12)	14 (23.7)	
Phase Ⅱ/Ⅲ	2 (1.7)	0 (0)	1 (1.7)	
Phase Ⅲ	24 (20.5)	19 (76)	36 (61)	
Phase Ⅳ	0 (0)	1 (4)	7 (11.9)	
Randomization				
Nonrandomized	79 (67.5)	6 (24)	21 (35.6)	<0.001
Randomized	38 (32.5)	19 (76)	38 (64.4)	
Blinding				
Double-blind	14 (12)	10 (40)	16 (27.1)	0.002
Open-label	103 (88)	15 (60)	43 (72.9)	
Comparator				
Active comparator	14 (12)	11 (44)	21 (35.6)	<0.001
Placebo	12 (10.3)	8 (32)	14 (23.7)	
None	91 (77.7)	6 (24)	24 (40.7)	
Multicenter				
Yes	62 (53)	12 (48)	18 (30.5)	0.018
No	55 (47)	13 (52)	41 (69.5)	
Chinese mainland population				
Included	79 (67.5)	25 (100)	59 (100)	<0.001
Not Included	38 (32.5)	0 (0)	0 (0)	
Primary study endpoints				
RR	92 (78.7)	6 (24)	17 (28.8)	<0.001
PFS	6 (5.1)	13 (52)	23 (38.9)	
OS	7 (6)	2 (8)	8 (13.6)	
OS + PFS	2 (1.7)	0 (0)	2 (3.4)	
OS + RR	2 (1.7)	0 (0)	0 (0)	
MFS	1 (0.8)	0 (0)	0 (0)	
Other Endpoints	7 (6)	4 (16)	9 (15.3)	

Note: P* value was calculated based on Kruskal–Wallis tests.

P values were calculated based on chi-square tests or Fisher’s exact test.

The median enrollment number of patients in pivotal premarketing clinical trials, completed postmarketing clinical trials, and ongoing postmarketing clinical trials was found to be 107 (IQR: 61–221), 289 (IQR:114–455), and 161 (IQR: 65–332), respectively. Pivotal premarketing clinical trials exhibited a significantly lower median enrollment of patients compared to completed and ongoing postmarketing clinical trials (p < 0.001).

Regarding the features of clinical trials, there was a statistically significant difference in the utilization of single-arm trials among pivotal premarketing clinical trials (77.8%) compared to completed postmarketing clinical trials and ongoing postmarketing clinical trials (24% and 39%, respectively; p < 0.001). We observed a significant disparity in the stage of clinical trials (p < 0.001). The stages of pivotal premarketing clinical trials were primarily designed as Phase II (51.3%), followed by Phase I/II (21.4%), Phase III (20.5%), and Phase I (5.1%). Conversely, most postmarketing clinical trials were categorized as Phase III (completed: 76%, ongoing: 61%). Furthermore, a notable increase in randomization was identified in postmarketing clinical trials compared to pivotal premarketing clinical trials (p < 0.001). The blinding designation of the pivotal and postmarketing clinical trials exhibited differences, with a higher prevalence of open-label labels observed in pivotal premarketing trials compared to postmarketing trials (88% vs. 60% for completed trials, and 72.9% for ongoing trials, p = 0.002). Most pivotal premarketing clinical trials (77.7%) lacked comparators, with only 12% utilizing active comparators and 10.3% employing placebo controls. Postmarketing clinical trials exhibited a notable rise in the utilization of active comparators (completed: 44%, ongoing: 35.6%). 53% of the pivotal premarketing clinical trials were conducted as Multi-Region Clinical Trials (MRCTs), with a decrease in the proportion of MRCTs in postmarketing clinical trials (completed:48%, ongoing: 30.5%; p = 0.018). A total of 67.5% (79/117) of the pivotal premarketing clinical trials for CAs included participants from mainland China, whereas all postmarketing clinical trials incorporated the Chinese mainland population (p < 0.001). Among the pivotal premarketing clinical trials, 117 primary endpoints were assessed. Most of these trials (96.6%) concentrated on a single primary endpoint, whereas a minority (3.4%) incorporated multiple primary endpoints. Specifically, the primary endpoint was RR in 78.7% of cases, OS in 6%, other endpoints in 6%, PFS in 5.1%, OS combined with PFS in 1.7%, and OS combined with RR also in 1.7% (Table 2). Postmarketing clinical trials should strive to enhance the level of evidence that confirms the effectiveness of drugs (Li et al., 2023). Consequently, postmarketing clinical trials exhibited significantly greater PFS and OS utilization than pivotal clinical trials (p < 0.001).

#### 3.1.3 The commencement time and length of postmarketing clinical trials

We defined the commencement of the postmarketing clinical trial as the enrollment of the first subject. As indicated in Table 3, 57.1% (48/84) of postmarketing clinical trials were commenced before (including on) the submission of CA, while the remaining 42.9% (36/84) were initiated after that. In the context of drug classification, the commencement of confirmatory trials for chemical drugs commonly occurred before the submission of applications for CAs (p < 0.001). This sequence of events led to a substantial proportion of completed conversions being attributed to chemical drugs (68%, 17/25). Completed postmarketing clinical trials were more likely to initiated before the submission of conditional applications, in contrast to the ongoing postmarketing clinical trials (p < 0.001). No discernible variances were observed regarding antineoplastics or non-antineoplastics agents, domestic or imported, or original indication approvals versus supplemental indication approvals at the commencement of postmarketing clinical trials.

**TABLE 3:** Differences in the initiation time of postmarketing clinical trials and the postmarketing clinical trials restriction period for CAs by NMPA, 2020–2023.

Variables	Studies initiated before the submission (n = 48) No. (%)	Studies initiated after the submission (n = 36) No. (%)	Postmarketing clinical trials restriction period (n = 49) Median (IQR, days)	p^a^	p^b^
Drug type					
Chemical drugs	34 (70.8)	12 (33.3)	1,460 (1,095,1825)	<0.001	0.076
Therapeutic biological products	14 (29.2)	23 (63.9)	1825 (1,551.25,1825)		
Traditional Chinese medicines	0 (0)	1 (2.8)	1,451		
Therapeutic area					
Antineoplastics agents	46 (95.8)	33 (91.7)	1825 (1,095,1825)	0.424	0.248
Non-antineoplastics agents	2 (4.2)	3 (8.3)	1825		
Indication approval sequence					
Original indication approvals	37 (77)	21 (58.3)	1825 (1,095,1825)	0.066	0.349
Supplemental indication approvals	11 (23)	15 (41.7)	1825 (1,460,1825)		
Origin					
Imported	24 (50)	16 (44.4)	1825 (1,460,1825)	0.614	0.033
Domestic	24 (50)	20 (55.6)	1,460 (1,095,1825)		
Trial category					
Completed	23 (47.9)	2 (5.6)	1,460 (1,095,1825)	<0.001	<0.001
On-going	25 (52.1)	34 (94.4)	1825 (1,460,1825)		

Note.

^a^ We conducted the chi-square test to assess differences in the initiation time of confirmatory studies across variables including drug type, therapeutic area, indication approval sequences, origin, and trial category.

^b^ We also performed the Mann-Whitney U or the Kruskal–Wallis test to assess differences in the postmarketing clinical trials restriction period by the above variables.

Among the postmarketing clinical trials, 49 were constrained by time limitations from the NMPA in fulfilling postmarketing obligations. We found that the median postmarketing clinical trials restriction period for the imported drugs was 1825 days (IQR:1,460–1825), which was notably longer than the median restriction period of 1,460 days (IQR:1,095–1825) for domestic drugs (p = 0.033). Furthermore, our study revealed that approvals that had transitioned to regular status exhibited shorter time restrictions than those that are still ongoing (p < 0.001). The median restriction period for transition to RA was 1,460 days (IQR: 1,095–1825), significantly shorter than 1825 days (IQR:1,460–1825) for ongoing approvals. Conversely, we found no notable differences in the postmarketing requirement restrictions based on drug type, therapeutic area, or indication approval sequence, as indicated in Table 3.

Out of the 25 approved conversions, CAs took a median of 826 days (IQR: 588–939) to go from conditional to regular approval, significantly shorter than the median restriction period of 1,460 days (IQR: 1,095–1825) (p < 0.001). The mean duration of the required restriction time exceeded the actual completion time, with respective averages of 1,357.8 days and 769.41 days. The maximum actual completion time observed was 1,060 days, while the minimum was 42 days. In contrast, the maximum required restriction time was 1825 days, with a minimum of 336 days (Supplementary Table S6, see Supplementary Material).

#### 3.1.4 Postmarketing obligations

The NMPA mandated 152 postmarketing obligations for the CAs listed between 2020 and 2023.93 (61.2%) obligations focused on verifying both the efficacy and safety of the drug. 7 (4.6%) and 5 (3.3%) obligations were dedicated to individually determining the efficacy or safety of the drug. Furthermore, 16 (10.5%) obligations were intended to complement the findings of pharmacokinetic studies, encompassing aspects such as drug interactions, dose optimization, and dose determination. The remaining 31 (20.4%) obligations encompassed promptly updating drug insert information and implementing a postmarketing risk management program.

### 3.2 Clinical evidence

#### 3.2.1 Efficacy evidence

This study revealed that a significant proportion of CAs pertained to antineoplastic indications. Consequently, an investigation was undertaken to evaluate the clinical evidence, using antineoplastic approvals as a case study. We conducted a meta-analysis for the primary effectiveness outcomes of antineoplastics approvals, including HR for PFS, OS in RCTs, and RR for single-arm trials or the RCTs.

The median RR observed in single-arm trials utilized for the CAs was 58% (IQR: 35%–73%). The range of RR varied from 13% to 92%. The pooled RR was 55% (95% CI: 49%, 60%; I^2^ = 95%). The median DOR of single-arm trials was 10.7 months (IQR:7.85–16.70) (Table 4). We conducted a subgroup analysis to investigate the magnitude of RR and the median DOR concerning the origin of drugs, drug types, indication approval sequences, and cancer types (Supplementary Figure S1, see Supplementary Material). The findings indicated a statistical variance in the pooled RR between chemical drugs and therapeutic biological products, with chemical drugs exhibiting a higher RR of 62% compared to 45% for therapeutic biological products (P = 0.001). There was a significant difference in cancer types (P < 0.001), with the highest pooled RR of ovarian cancer indication (69%), followed by thyroid cancer and lymphoma (all 66%). Similarly, a significant difference also in the median DOR of cancer types (P = 0.011). The most prolonged median DOR for the treatment of solid cancer was 34.50 months, followed by cervical cancer (20.11 months) and thyroid cancer (17.50 months).

**TABLE 4:** Pivotal premarketing clinical trial outcomes of CAs by NMPA, 2020 to 2023.

Clinical evidence	
Efficacy	
Response rate (single-arm trials)	
Median,%, (IQR)	58 (35–73)
Pooled estimate,%, (IQR)	55 (49–60)
Duration of Response, months, median (IQR)	10.70 (7.85–16.70)
Progression-free survival (RCTs)	
Gain, months, median (IQR)	14.9 (7.7–20.67)
Pooled hazard ratio (IQR)	36 (24–48)
Improvement, median (IQR)	6.8 (5.3–9.6)
Overall survival (RCTs)	
Gain, months, median (IQR)	12.97 (10.05–18.07)
Pooled hazard ratio (IQR)	68 (61–73)
Improvement, median (IQR)	3.98 (3.77–6.32)
Response rate (RCTs)	
Median,%, (IQR) (The investigational group)	42 (36–95)
Median,%, (IQR) (The control group)	19.5 (16.7–53.5)
Pooled relative risk (95%CI)	1.90 (1.61–2.26)
Safety	
Single-arm trials	
SAEs, pooled estimate (95%CI)	26 (22–30)
Grade ≥3 AEs, pooled estimate (95%CI)	44 (40–49)
RCTs	
SAEs, pooled relative risk (95%CI)	1.20 (0.96–1.50)
Grade ≥3 AEs, pooled relative risk (95%CI)	1.09 (0.89–1.35)

The pooled HR for PFS was 36% (95% CI: 24%, 48%; I^2^ = 88.6%), while the pooled HR for OS was 68% (95% CI: 61%, 73%; I^2^ = 0%). The median PFS duration for CAs was 14.9 months (IQR, 7.7–20.6), and the median OS duration was 12.97 months (IQR, 10.05–18.07). In comparison to the control group in RCTs, there was an increase of 6.8 months (IQR, 5.3–9.6) in the median PFS and 3.98 months (IQR, 3.77–6.32) in the median OS, respectively (Table 4). Concerning the drug origins, drug types, indication approval sequences, and cancer types, there was no significant difference in the PFS or OS improvement time (Supplementary Figures S2, S3; see Supplementary Material).

In the context of RCTs, RR was assessed in six approvals. The median RR in the RCTs was different. It was 42% (IQR: 36%–95%) in the investigational group, whereas 19.5% (IQR: 16.7%–53.5%) in the control group. The experimental group demonstrated a significantly higher RR compared to the control group, with a relative risk of 1.90 (95%CI: 1.61–2.26, P < 0.001) (Supplementary Figure S4, see Supplementary Material).

Sensitivity analysis indicated that the combined RR, PFS, and OS estimates remained robust even after excluding individual studies. Egger’s and Begg’s tests for the RR in the single-arm trials with p-values were 0.18 and 0.376, respectively; in the RCTs, the p-values were 0.427 and 0.26, respectively. Egger’s test p-values for the pooled PFS and OS were 0.22 and 0.582, and Begg’s test p-values for them were 0.703 and 0.076, respectively. Therefore, there was no evidence of publication bias.

#### 3.2.2 Safety evidence

The pooled relative risk of SAEs and Grade≥3 AEs were 26% (95% CI:22%–30%, I^2^ = 96.31%) and 44% (95% CI: 40%–49%, I^2^ = 97.83%), respectively, in single-arm trials (Table 4). There was no significant difference in the incidence of SAEs (relative risk = 1.20, 95% CI: 0.96–1.50, P = 0.051) and Grade ≥3 AEs (relative risk = 1.09, 95% CI: 0.89–1.35, P = 0.29) between the investigational group and their corresponding controls in the RCTs. (Supplementary Figures S7, S8, Supplementary Figures S5, S6, see Supplementary Material).

Sensitivity analysis confirmed the reliability of the pooled SAEs and Grade ≥3 AEs results even after excluding individual studies. The combined findings from RCTs indicated no publication bias for SAEs and Grade ≥3 AEs (P = 0.52 and P = 0.679 for Egger’s test, respectively; P = 0.964 and P = 0.464 for Begg’s test, respectively). Regarding the single-arm trials, no publication bias was detected for Grade ≥3 AEs (P = 0.925 for Egger’s test; P = 0.785 for Begg’s test), whereas the SAEs observed publication bias (p < 0.05 for Egger’s and Begg’s tests).

### 3.3 Implementation effects

#### 3.3.1 CA can significantly reduce drug development time, review time, and drug lag period

We conducted a comparative analysis of the development time, review time, and drug lag periods between CAs and non-CAs to assess the implementation effects of the CAs for drugs in China. Given that the majority of CAs were for antineoplastics agents, aligning with the general trend in CAs, we specifically analyzed CAs for antineoplastics agents from 2020 to 2023, with non-CAs for antineoplastics agents serving as the control group during the same timeframe. A total of 178 approvals for antineoplastics agents were identified from 2020 to 2023, with 88 (49.4%) granted CAs and 90 (50.6%) classified as non-CAs (Supplementary Table S7, see Supplementary Material online). Drug development time was defined as the duration between IND approval and NDA submission, and drug review time was defined as the duration from NDA submission to NDA approval. For imported drugs approved via a bridging study, the development timeline was determined starting from the date of bridging study approval. Conversely, for imported drugs exempted from clinical studies in China, the development timeline was considered to be zero.

**FIGURE 2:**
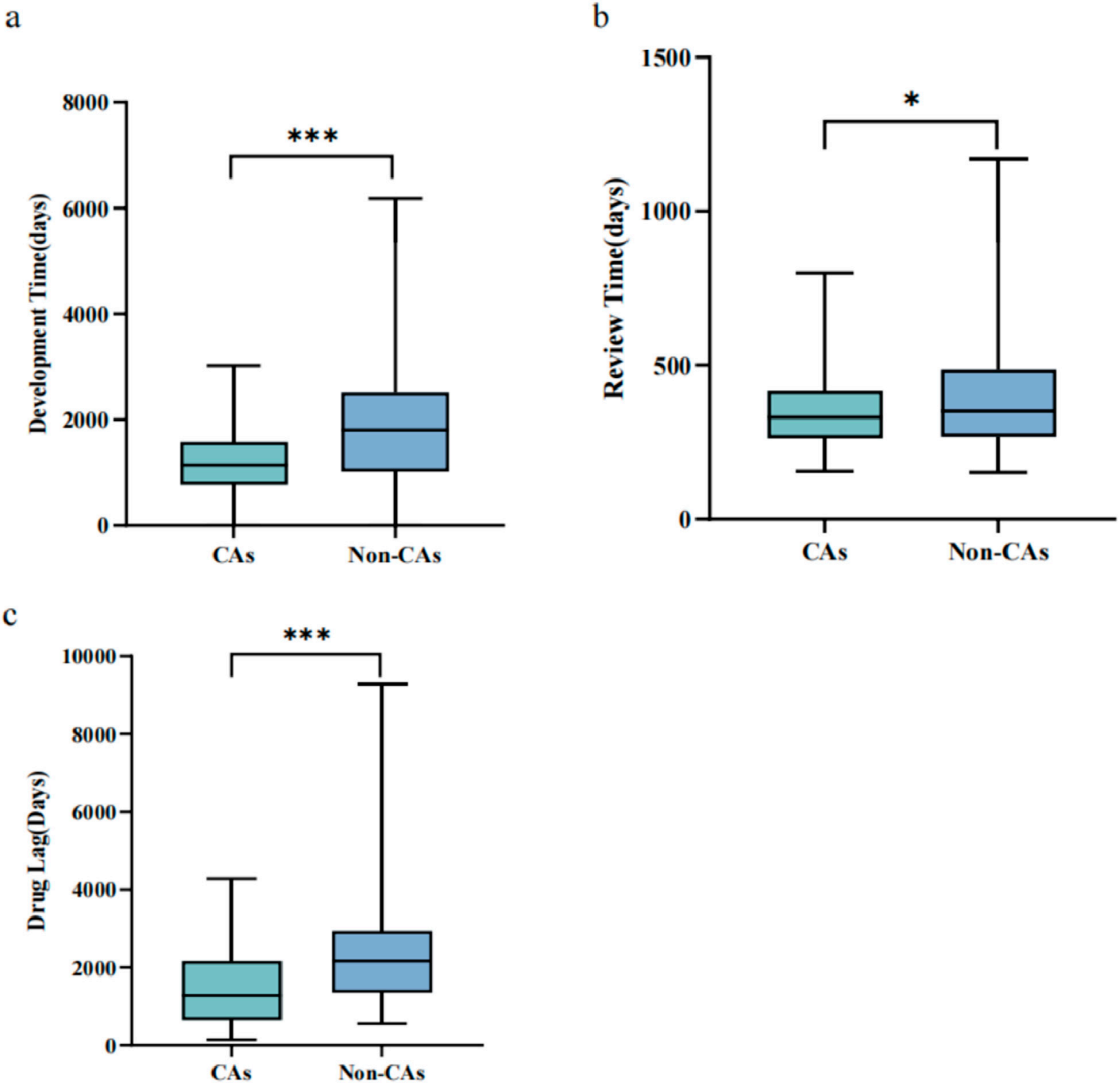
The implementation effects of the CA for the antineoplastic agents, 2020–2023 **(A)** Development time comparison between CAs and non-CAs for antineoplastic agents **(B)** Review time comparison between CAs and non-CAs for antineoplastic agents **(C)**. Drug lag period comparison of the antineoplastics agents between CAs and non-CAs. Box plots display the interquartile ranges through shaded areas and depict the maximum and minimum values with whiskers. *p < 0.05, ***p < 0.001.

The approval pathway significantly influenced the duration of drug development, as illustrated in Figure 2A. The median development time for CAs was notably shorter than non-CAs (1,139.5 vs. 1804.5 days, p < 0.001). On average, CAs had a shorter development time than non-CAs (1,137.3 vs. 1903.7 days). The maximum development time for CAs was 3,021 days, while non-CAs had a maximum development time of 6,177 days.

Similar results were observed in the drug review time for antineoplastics agents from 2020 to 2023. The median review time for CAs was 331 days (IQR: 261.5–417.75 days), compared to 380 days (IQR: 282–576 days) for non-CAs, which had significant statistical differences (P = 0.032) (Figure 2B). Additionally, the average review time for CAs was shorter than non-CAs (356.69 days vs. 408.76 days). Furthermore, the maximum review time for CAs was 800 days, whereas the maximum for non-CAs was 1,171 days.

We also conducted an analysis to investigate the drug lag period of the antineoplastics agents. Of the 178 antineoplastics approvals examined, 101 (56.7%) were approved in both China and other countries, consisting of 4 initially approved in China and 97 initially approved in other countries. For analysis, we specifically focused on the 97 approvals initially approved in other countries, encompassing 45 CAs and 52 non-CAs. We utilized the original approval dates for antineoplastics approvals globally as a benchmark for assessing the lag in the availability of these agents in China. A total of 52 non-CAs were selected as the control group. The study found that the drug lag period for CAs was significantly shorter compared to non-CA drugs, as evidenced by the median drug approval time (p < 0.001) (Figure 2C).

#### 3.3.2 Differences exist in development time, review time, and drug lag period among CAs

To further investigate the differences in development time, review time, and drug lag period among CAs, we performed a subgroup statistical analysis considering various factors: drug origins (imported or domestic), drug types (therapeutic biological products or not), indication approval sequences (original or supplemental indication approval), inclusion in PR, and inclusion in BTD.

**FIGURE 3:**
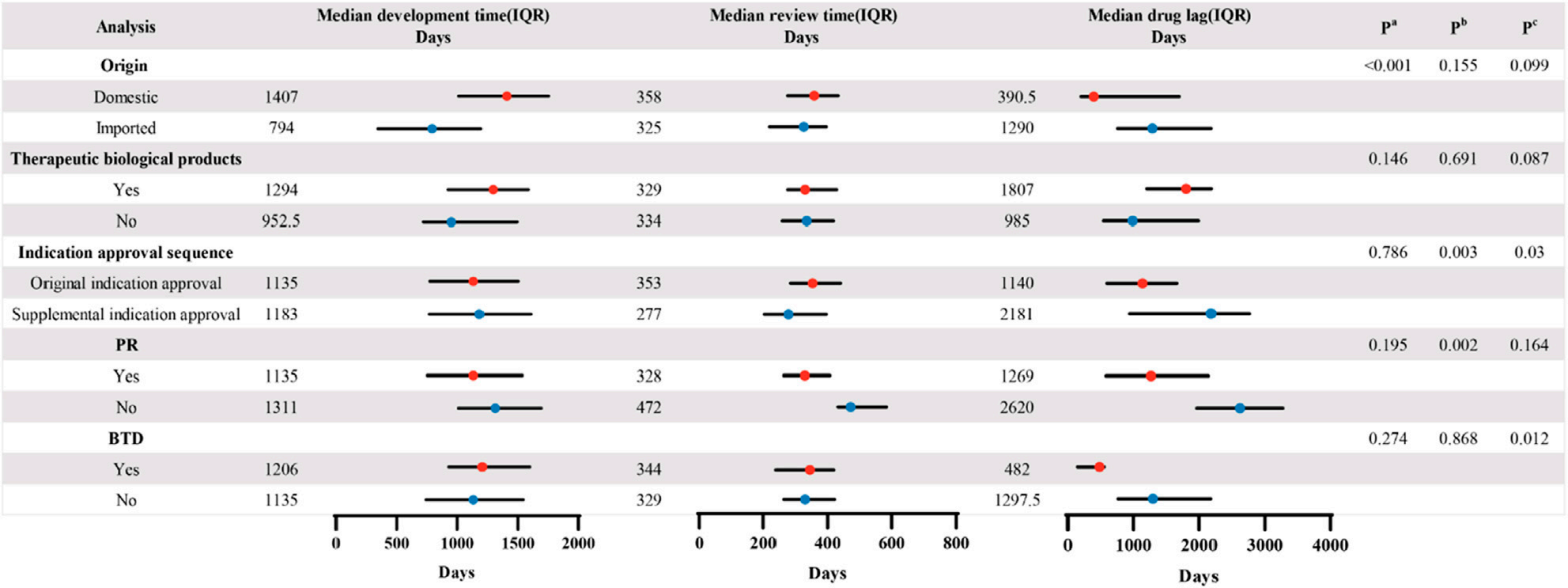
Comparison of the CAs’ median development time, review time, and drug lag period in the different subgroups. P values were calculated based on the Mann–Whitney U test in the two groups. P^a^. Comparison of median development time in different variables. P^b^. Comparison of median review time in different variables. P^c^. Comparison of median drug lag period in different variables.

As depicted in Figure 3, the data revealed a significant disparity in development time among drugs of different origins. The median development time for imported drugs was 794 days, markedly shorter than the median development time for domestic drugs, which was 1,407 days (p < 0.001). Regarding review time, statistically significant differences were observed between the two subgroups based on the sequence of indication approvals and their inclusion in the PR. The median review time for the supplemental indication approval was significantly shorter than that for the original indication approval (277 vs. 353 days, p = 0.003). Inclusion in PR significantly reduced the review time (328 vs. 472 days, p = 0.002). We also found that the drug lag period was statistically different for the indication approval sequences and the inclusion of BTD. The results demonstrated that the median drug lag period for the original indication approvals was significantly shorter than that for the supplemental indication approvals (1,140 vs. 2,181 days, p = 0.03). Furthermore, the CAs that were granted BTD also appeared to reduce the median drug lag period in comparison to non-BTD drugs (482 vs. 1,297.5 days, p = 0.012).

Specifically, clinical development strategies were the principal factor contributing to the shorter development time of imported drugs than domestic drugs. The main clinical development strategies for drugs marketed in mainland China include MRCTs, local clinical trials, bridging, and waiving. In 2017, NMPA joined the International Council for Harmonization of Technical Requirements for Pharmaceuticals for Human Use (ICH) while adopting a more accommodating and receptive stance towards the utilization of overseas clinical trial data (National Medical Products Administration, 2024c). In 2018, the CDE issued the Technical Guidelines for Accepting Overseas Clinical Trial Data of Drugs, which allowed for the full or partial acceptance of clinical trial data from overseas that meet the criteria of authenticity, completeness, accuracy, and traceability (National Medical Products Administration, 2018). Consequently, a considerable number of imported drugs have gained access to the market in China through MRCTs, bridging or waiving, thereby significantly accelerating their development. Furthermore, to expedite the approval of urgently needed overseas drugs (UNODs) in China, the NMPA has initiated the review and approval of new drugs outside of China for urgent clinical needs and released the three lists of Batches of UNODs in Clinical Settings (National Medical Products Administration, 2020c). The listed drugs can apply for the class I communication meeting with the CDE, and are eligible for rolling review, PR, and exemptions from clinical trials and other supportive policies, thereby significantly accelerating the research and development process. Among the CAs for imported drugs, 4 were for drugs identified as UNODs with clinical study exemptions, which including Atezolizumab, Nivolumab, Pembrolizumab, and Ipilimumab. More than 97.3% CAs for imported drugs were based on MRCTs, bridging, or waiving. In contrast, the majority of CAs for domestic drugs (65.5%) were approved through local clinical trials, resulting in a longer development timeline.

Regarding the review time, the standard review timeframe for drugs granted PR will be shortened from 200 working days to 130 working days. If drugs for rare diseases are also included in UNODs lists, their review time will be reduced to 70 working days (Zou et al., 2023). Therefore, inclusion in the PR is an essential factor affecting the review time. In addition, The assessment of supplementary indication applications primarily concentrates on the efficacy of the newly incorporated indications. The evaluation of pharmacokinetics, pharmacodynamics, and safety is predicated on pre-existing evidence. Consequently, this approach results in a substantially reduced median review duration for supplementary indications relative to the median review time for the original indications.

In China, the BTD program offers a range of supportive policies, including rolling review, eligibility for Class I communication meetings, and technical guidance during the development phase. This program essentially ensures automatic PR designation, thereby expediting the launch of BTD drugs and mitigating delays in drug availability (Luo et al., 2022).

Similarly, the original indications demonstrate greater innovation compared to supplementary indications, as evidenced by their higher likelihood of being granted BTD (11/65 vs. 4/30, 16.9% vs. 13.3%) and PR (59/65 vs. 26/30, 90.8% vs. 86.7%). Consequently, original indications experience a shorter drug lag relative to supplementary indications.

#### 3.3.3 Unmet clinical needs and public health emergency can be partially addressed through

##### 3.3.3.1 The implementation of CAs

Out of the 103 CAs examined, 17 (16.5%) demonstrated a significant improvement in disease prognosis compared to existing treatments. Additionally, 22 approvals (21.4%) were utilized to augment the effectiveness of available therapies for patients who were either intolerant or unresponsive to those treatments. Furthermore, 4 approvals (3.9%) were found to effectively mitigate the serious adverse effects associated with existing treatments. Additionally, 20 approvals (19.4%) were approved for the first time to address unmet medical needs, while 32 (31.1%) approvals did not have standard available treatments before receiving CA. Moreover, 8 approvals (7.7%) were used to treat COVID-19, addressing the urgent need for public health (Supplementary Table S1, see Supplementary Material).

## 4 Discussion

This study sought to thoroughly evaluate the characteristics, clinical evidence, and implementation effects of the CAs granted in China since the formal introduction of the CA program. Our findings indicated that 103 indications were conditionally approved from 2020 to 2023. These approvals spanned various therapeutic categories, such as antineoplastic, blood and blood-forming organs, immunomodulating, anti-infective for systemic use, dermatological, and musculoskeletal, demonstrating the CA program’s regulatory adaptability in addressing severe and life-threatening indications (Luo et al., 2023). The CA program has the potential to decrease review time, development time, and drug lag duration to expedite drug approval and has significantly improved the accessibility of urgently needed medications, thereby playing a crucial role in safeguarding public health. Nonetheless, the CA program in China may encounter specific challenges.

In comparison to the Accelerated Approvals (AAs) of the United States Food and Drug Administration (FDA) and the Conditional Marketing Approvals (CMAs) of the European Medicines Agency (EMA), the distribution of indications for CAs in China exhibits a higher degree of concentration, predominantly in antineoplastics agents. There are some reasons for this result. Initially, the NMPA has enacted policy incentives through the issuance of a series of guidelines pertaining to antineoplastic medicines. Significantly, these guidelines endorse the utilization of single-arm trial data for the approval of antineoplastic medicines, to enable such drugs to undergo pivotal single-arm trials grounded in robust scientific evidence. These initiatives not only enhance the efficiency of research and development processes but also promote more effective and targeted communication between applicants and the CDE. Moreover, certain pharmaceutical companies are motivated by the objective of increasing their profitability. Recent data from the World Health Organization indicate that China exhibits the highest incidence and mortality rates of cancer worldwide, signifying that cancer constitutes the predominant health burden in the country (Luo et al., 2023). As a result, some pharmaceutical companies are increasingly focusing on the antineoplastic drug market. Furthermore, the Conditional Approval program for drugs in China is pertinent in scenarios where a drug is designed to address life-threatening diseases lacking existing curative treatments or in instances where there is an urgent public health necessity. Antineoplastic medicines are particularly well-suited to these criteria. It is recommended that the NMPA enhances policy support, technical guidance, economic assistance, and other relevant dimensions to expand the CA coverage.

Pivotal premarketing clinical trials commonly utilized single-arm trials to support CAs. The absence of randomization and parallel control groups in single-arm trials may introduce bias, thereby constraining their capacity to accurately assess the risk-benefit profile. In March 2023, FDA published draft guidance entitled “Clinical Trial Considerations to Support Accelerated Approval of Oncology Therapeutics Guidance for Industry” (Food and Drug Administration, 2023). It is recommended that RCTs be utilized as the preferred method to substantiate an application for accelerated approval. The “Technical Guidelines on the Applicability of Single-Arm Clinical Trials for Use in Support of Oncology Drug Marketing Applications” issued by the CDE of NMPA in March 2023 underscores the viability of single-arm trials in exceptional circumstances where conducting RCTs is challenging. Additionally, the NMPA has put forth various technical guidelines regarding the suitability of single-arm trials in the context of marketing applications for CA, explicitly delineating the constraints associated with single-arm trials (National Medical Products Administration, 2023b). Consequently, it is essential to comply with pertinent regulatory guidelines to use single-arm studies to support CAs. More importantly, incorporating the utilization RCTs as much as possible. We observed a significant increase in the use of RCTs in the design of postmarketing clinical trials, especially in the completed postmarketing clinical trials. Up to 76% completed postmarketing clinical trials utilized RCTs, with 15 of them transited from premarketing single-arm trials to postmarketing RCTs. This means that postmarketing clinical trials have provided stronger evidence support and are more reliable to verify the benefit of the drugs.

In 2002, the Chinese government initially allowed overseas applicants to conduct MRCTs in China, but restricted this to drugs that were either already registered outside China or in phase II or III of clinical trials. In 2015, this policy was expanded to include new drugs not yet approved overseas, facilitating synchronous clinical trials and encouraging the participation of domestic clinical trial institutions in MRCTs. In 2020, the CDE issued the “Clinical Technical Requirements for Overseas Listed and Domestic Unlisted Drugs,” which further incentivizes pharmaceutical companies to conduct clinical trials in China concurrently through MRCTs (National Medical Products Administration, 2018). In pivotal premarketing clinical trials, 53% were conducted via MRCTs, with domestic and imported products comprising 27.4% and 72.6%, respectively. These findings indicated that MRCTs had played a significant role in premarketing clinical trials supporting CAs. However, there is a lack of regulations pertaining to MRCTs for postmarketing research, and the NMPA mandates postmarketing confirmatory trials to specifically validate safety and efficacy in Chinese patients. So the number of MRCTs declined to 30 in the postmarketing research stage. Moreover, due to the marketing delays of CAs in China relative to other countries, numerous imported drugs have commenced confirmatory trials in foreign nations prior to receiving approval in China (Wei et al., 2024). Consequently, MRCTs are rendered unnecessary for postmarketing research within China. Analysis of the data revealed that there were 45 MRCTs for imported drugs at the premarketing research stage; however, only 18 of these continued as MRCTs in the postmarketing phase. Additionally, although nearly half of CAs are consisted of approvals for domestic drug, but only 4 approvals have marketed overseas. These conducted MRCTs in the premarketing research stage might convert to domestic clinical trials for the failure of marketing overseas. The data indicated that there were 17 MRCTs for domestic drugs at the premarketing research stage; however, only 2 of these continued as MRCTs in the postmarketing phase. From the analysis above, we can conclude that despite the substantial advancements in CAs in China, the marketing delay of the imported drugs and the constraints faced by domestic drugs in achieving global reach remain critical issues that warrant attention and these also lead to the decrease of MRCTs in the postmarketing trials.

Our study found a significant prevalence of surrogate endpoint utilization in pivotal and confirmatory trials. While surrogate endpoints are often employed to shorten the clinical trial duration and accelerate the launch of life-threatening drugs, particularly in the realm of oncology, the validity of their translation into patient clinical benefit is a subject of ongoing debate (Salcher-Konrad et al., 2020; Gyawali et al., 2021). Several recent studies have shown a weak correlation between surrogate endpoints and clinical benefit, highlighting the importance of carefully selecting appropriate surrogate endpoints when developing new drugs (Schuster Bruce et al., 2019; Kim and Prasad, 2016; Haslam et al., 2019). Currently, China has not established guidelines or systems for the development and identification of surrogate endpoints or published a list of such endpoints (Yang et al., 2022). Drawing on the experiences of regulatory bodies such as the FDA and the EMA in utilizing surrogate endpoints, it is recommended that the China NMPA establish protocols for the development and identification of surrogate endpoints (US Food and Drug Administration, 2022). Moreover, it must be acknowledged that the creation of a catalog of recognized surrogate endpoints would contribute to improving the efficacy of drug approval.

In our study, RR emerged as the primary endpoint in pivotal premarketing clinical trials of CAs, accounting for 78.7%. Cherny NI et al. analyzed the 58 AAs that employed single-arm trials with RR endpoints by the FDA, revealing a median RR of 40% (Cherny, 2022). Furthermore, CAs in China demonstrated a notably higher median RR of 58% (IQR: 35%–73%) in comparison to FDA approvals based on single-arm trials. Moreover, our analysis revealed notable variations in RR among drug types and cancer types, indicating the challenge in establishing a universal criterion for RR. Specifically, the highest pooled RR was observed in ovarian cancer at 69%, contrasting with the lowest rate of 14% in liver cancer. Additionally, our findings indicated a pooled RR of 62% for chemical drugs compared to 45% for therapeutic biological products. As a result, it is advisable to consider a flexible approach in determining RR thresholds for surrogate endpoints.

Our research findings indicated that 21 CAs based on RR had converted to regular approval, with a median RR of 63% (IQR: 33%–70%). This observation aligns with previous assertions and suggests that high RR values (>60%) may provide stronger evidence of the clinical benefit (Cherny, 2022). In pivotal single-arm trials, the RR of primary endpoints exhibited significant variability, ranging from 13% to 92%, with 4 drugs (4.7%) demonstrating an RR below 20%. A previous data analysis discovered that 81% (21 out of 26) of cancer indications revoked by the FDA had been evaluated in single-arm trials, with an RR below 24% (Chen et al., 2019). It is necessary to establish a baseline requirement for RR, as applications failing to meet this threshold will not receive approval.

The safety profile of approved drugs is a notable concern in the context of the CA program. This study aimed to aggregate adverse events reported in pivotal clinical trials for CAs. The pooled data (26% for SAEs, 44% for Grade ≥3 AEs) from single-arm trials indicated that the safety risk associated with these drugs was manageable. Nevertheless, it is essential to note that most pivotal premarketing clinical trials for CAs were single-arm trials lacking parallel control groups, making it challenging to ascertain whether adverse events were attributable to the disease or the medication (Luo et al., 2024; Cucherat et al., 2020). Due to the absence of concurrent control groups in single-arm studies, these trials often depend on external controls, such as historical or real-world data, to provide comparative context. Consequently, it is imperative to ensure that the external control data underpinning the single-arm study of the drug are both transparent and reliable, facilitating a robust comparison with the study’s outcomes. Additionally, thorough monitoring of the safety data for the experimental drug during its initial phases is crucial, as is the evaluation of whether its safety profile justifies progression to subsequent clinical trials. Our analysis of the safety data of RCTs revealed no notable safety issues associated with CA drugs compared to the control group. However, it is crucial to recognize that these conclusions are drawn from a restricted sample size. Therefore, it is essential to expand the sample size for confirmatory trials and to persist in gathering safety data post-trial. Evidently, This effort has been demonstrated in the data from the postmarketing clinical trials from the analysis.

Variations in the timing of postmarketing confirmatory studies have been observed. Specifically, 42.9% of these studies were initiated after the submission for CA. Our analysis revealed that a notable proportion of drugs that converted to regular approval had already commenced their confirmatory studies before the submission for CA. It can be concluded that conducting confirmatory trials early in the process can significantly reduce the time gap between CA and the demonstration of clinical efficacy or lack thereof (Huang and Yuan, 2024). A study revealed that the median conversion duration for cancer drugs from accelerated to regular approval by the FDA was significantly shorter for approvals with confirmation trials that commenced before the submission of AA applications compared to those initiated after submission (3.1 years versus 5 years) (Huang and Yuan, 2024). This trend was also observed for non-antineoplastic agents with AA (Fashoyin-Aje et al., 2022). Therefore, it is advisable for pharmaceutical companies to commence confirmatory studies promptly. In the Consolidated Appropriations Act 2023, Congress provided FDA with additional authorities to require a confirmatory study or studies to be underway prior to approval. On 5 December 2024, the FDA issued draft guidance entitled “Expedited Program for Serious Conditions—Accelerated Approval of Drugs and Biologics” which stipulated that confirmatory testing must be “well under way” prior to receiving accelerated approval. Subsequently, on 6 January 2025, the FDA released another draft guidance, “Accelerated Approval and Considerations for Determining Whether a Confirmatory Trial is Underway”. The new guidance reaffirms the necessity for the initiation of drug confirmatory testing as a prerequisite for accelerated approval and provide detailed criteria for assessing whether such testing is being conducted effectively (US Food and Drug Administration, 2024; US Food and Drug Administration, 2025). The recently published guidance from NMPA, titled “Technical Guidance on the Suitability of Single-arm Clinical Trials to Support Marketing Applications for Cancer Drugs” emphasized the importance of initiating confirmatory clinical trials for antineoplastic agents before obtaining a CA (National Medical Products Administration, 2023b) Nonetheless, the formal enactment of this requirement necessitates explicit legal authorization and the formulation of accompanying regulations.

Our study revealed that a substantial portion of CAs lacked strict specifications for the timeline of the completion of confirmatory trials. Furthermore, a notable disparity was observed in the time constraints imposed on confirmatory trials by regulatory authorities based on the drug origin or trial category. Therefore, it is recommended that regulators adopt a more flexible approach in defining deadlines for confirmatory trials. Therapeutic areas should be a primary consideration. Among the 25 converted CAs, 24 pertain to antineoplastic indications, with a median duration of 687.5 days (IQR: 490.25–920.75) from CA to RA. However, for diseases where patient recruitment or endpoint achievement is challenging, the timeline for confirmatory trials should be extended. It is essential to establish a communication mechanism that allows applicants to discuss timelines with the NMPA before submitting a CA. Furthermore, enhancing the ongoing monitoring of confirmatory assessments is crucial to mitigate delays and prevent the prolonged availability of ineffective drugs in the market.

The research found that the NMPA review reports lacked a standardized format for postmarket obligations, which were broadly defined and lacked specific details regarding the requirements for confirmatory trials. This trend mirrors previous studies conducted on the FDA and the EMA, where the postmarketing requirements outlined in the FDA’s AA and the EMA’s CMA were only briefly described (Shahzad et al., 2023; Xie et al., 2023; Zhang et al., 2024). Therefore, regulators should improve the transparency of postmarketing trials for CAs by promptly updating relevant outcomes and providing a comprehensive description of the study design, trial endpoints, study population, and other necessary information. Additionally, sponsors and regulators should clearly label the National Clinical Trial (NCT) number or trial number for identified postmarketing studies to facilitate public inquiry and monitoring of their advancement.

Pharmacovigilance is particularly crucial under the conditional approval program, as these drugs are approved for marketing based on limited clinical data. However, the requirements for how sponsors should conduct specific pharmacovigilance activities after the drug granted CA in China are still unclear. China has not yet introduced specific regulations on post-marketing risk management and pharmacovigilance for conditionally approved drugs. Therefore, it is recommended that when submitting a CA application, applicants should clearly outline the specifics of the post-marketing risk management plan, including the details of how the sponsor will implement pharmacovigilance activities. Furthermore, it is advised that the NMPA of China should promptly establish dedicated regulations concerning post-marketing risk management and pharmacovigilance for conditionally approved drugs, offering explicit guidance to sponsors.

Currently, no conditionally approved drugs are withdrawn by NMPA from the market in China. Research indicated that certain accelerated approved drugs withdrawn by the FDA were presently approved in China or the investigational new drug phase (Chen et al., 2019). This discrepancy is attributed to the delays in the submission of CA applications in China compared to the US. This delay could result in the withdrawal of certain AA indications from the market in the United States, which are currently undergoing confirmatory trials or are still in regular use in China (Wei et al., 2024). Hence, enhancing collaboration and information exchange among regulatory agencies can reduce such disparities. Additionally, it is recommended that China promptly implement a withdrawal process for CA drugs lacking demonstrated clinical efficacy.

The CA program has been demonstrated to reduce drug development time, review time, and drug lag period. The subgroup analysis revealed that this effect would be further enhanced with concurrent BTD status, or PR status. It is evident that various expedited programs are applicable at distinct stages of the drug development process, thereby facilitating the advancement and introduction of drugs. Consequently, it is advisable to enhance the integration of BTD, PR, and CA pathways. The NMPA should more comprehensively illustrate the guidelines and regulations about the linkage of CA, BTD, and PR.

## 5 Limitations

Several limitations constrain our research. Firstly, a significant portion of drugs granted conditional approval have not yet undergone postmarketing confirmatory trials, resulting in a dearth of data in such trials. Secondly, when analyzing trial designs pre- and post-conditional approval in our study, it is essential to acknowledge that the data collected for ongoing confirmatory trials may not accurately reflect the actual trials conducted due to potential inconsistencies. Thirdly, the prevalence of conditionally approved antineoplastics agents in this study resulted in a disproportionate focus on these medications. Consequently, the extent to which the findings can be generalized to other therapeutic indications remains uncertain.

## Data Availability

The original contributions presented in the study are included in the article/Supplementary Material, further inquiries can be directed to the corresponding author.
