# Supplementary material for: Characteristics, clinical evidence and implementation effects of conditional approvals for drugs in China, a pooled analysis from 2020 to 2023
Source: Front Pharmacol. 2025 Apr 25;16:1501525. doi: 10.3389/fphar.2025.1501525 (PMC12061712; doi:10.3389/fphar.2025.1501525)
Supplement: Supplementary file 1 [file Supplementaryfile1.docx]

Supplementary Material

# Supplementary Figures


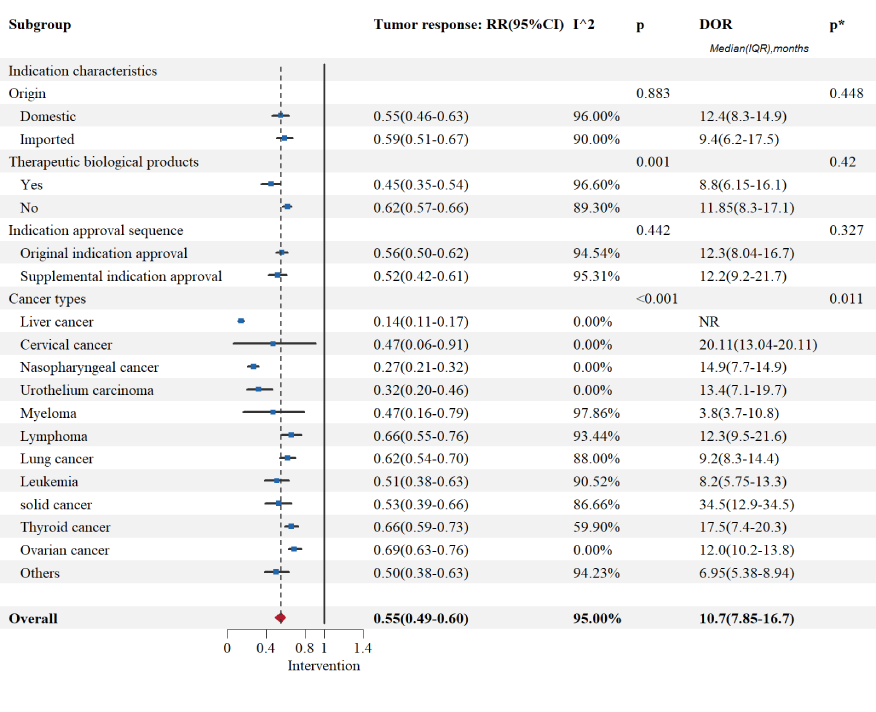


## Supplementary Figure 1. Subgroup meta-analysis of all single-arm trials reporting RR.

RR: Response rate; A single-arm study (dinutuximab beta) was excluded from the evaluation because it did not have a specified primary efficacy endpoint.The block and whiskers represent the weighted average and 95% CI, respectively. The diamond represents the pooled estimates. P calculated based on Cochran’s Q test. P* calculated based on Mann-Whitney tests or Kruskal-Wallis tests.

#
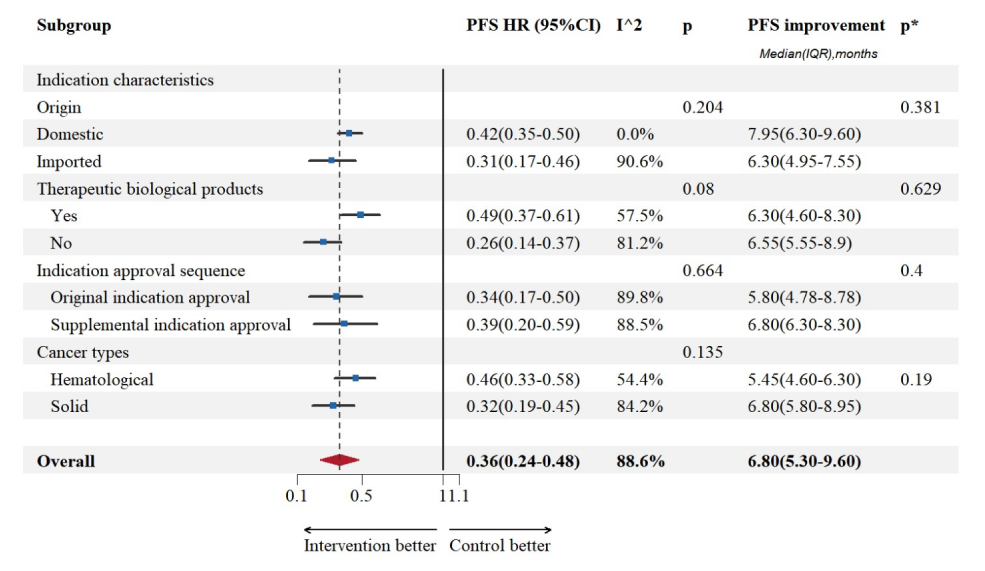


## Supplementary Figure 2.Subgroup meta-analysis of RCTs reporting PFS.

RCTs: Randomized controlled trials; PFS: progression-free survival. The block and whiskers represent the weighted average and 95% CI, respectively. The diamond represents the pooled estimates.

P calculated based on Cochran’s Q test. P* calculated based on Mann-Whitney tests.


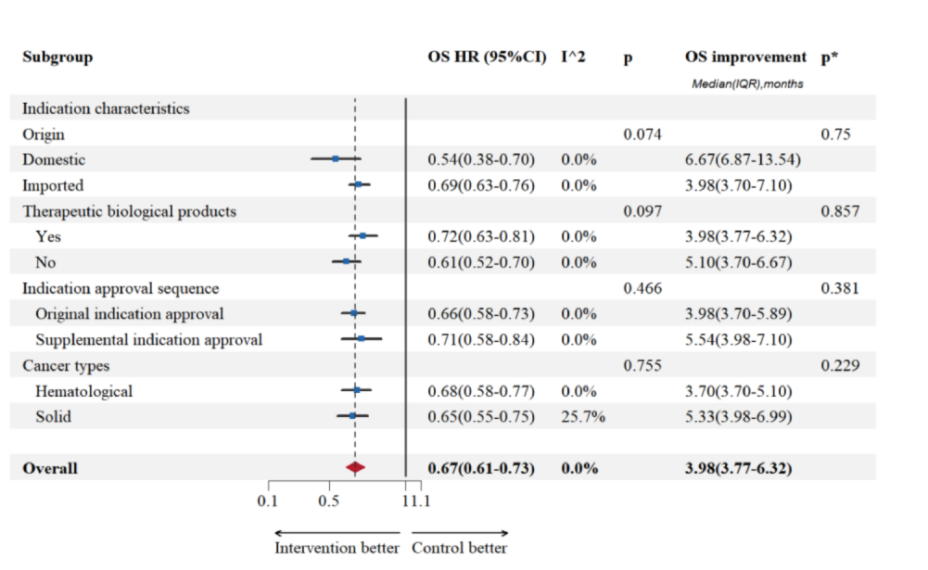


## Supplementary Figure 3. Subgroup meta-analysis of RCTs reporting OS.

RCTs: Randomized controlled trials; OS: overall survival. The block and whiskers represent the weighted average and 95% CI, respectively. The diamond represents the pooled estimates.

P calculated based on Cochran’s Q test. P* calculated based on Mann-Whitney tests.


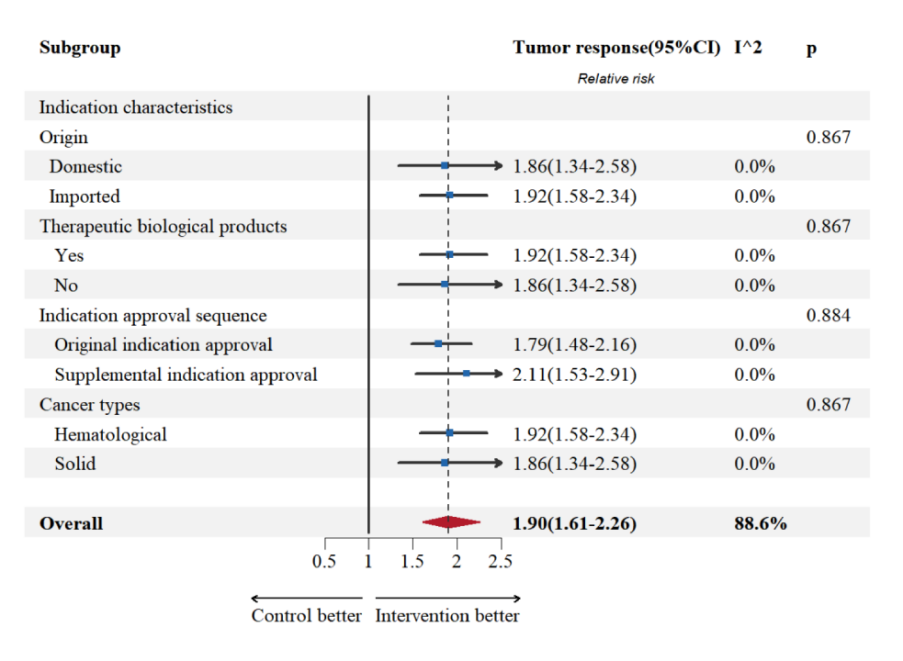


## Supplementary Figure 4.Subgroup meta-analysis of RCTs reporting RR.

RCTs: Randomized controlled trials; RR: Response rate; None of the results of the RCTs in which RR was the primary endpoint reached the median DOR. Therefore, no statistical analyses were performed on the improvement time to DOR in the RCTs. The block and whiskers represent the weighted average and 95% CI, respectively. The diamond represents the pooled estimates.

P calculated based on Cochran’s Q test.


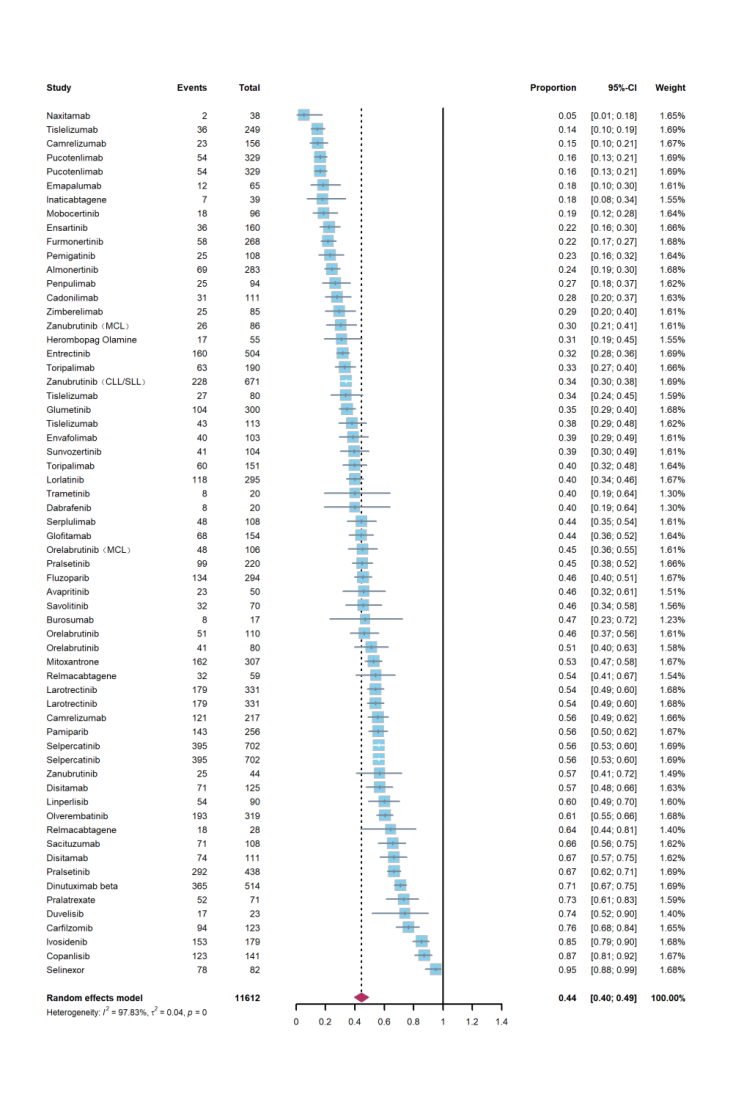


## Supplementary Figure 5. Forest plot of Grade ≥ 3AEs for single-arm pivotal trials for conditional approvals.

The block and whiskers represent the weighted average and 95% CI, respectively. The diamond represents the pooled estimates. The AEs level class according to the National Cancer Institute Common Terminology Criteria for AEs is as follows: Grade 1, Mild; asymptomatic or mild symptoms; clinical or diagnostic observations only; intervention not indicated. Grade 2, Moderate; minimal, local, or noninvasive intervention indicated; limiting age-appropriate instrumental activities of daily living (ADL). Grade 3, Severe or medically significant but not immediately life-threatening; hospitalization or prolongation of hospitalization indicated; disabling; limiting self-care ADL. Grade 4, Life-threatening consequences; urgent intervention indicated. Grade 5, Death related to AE.


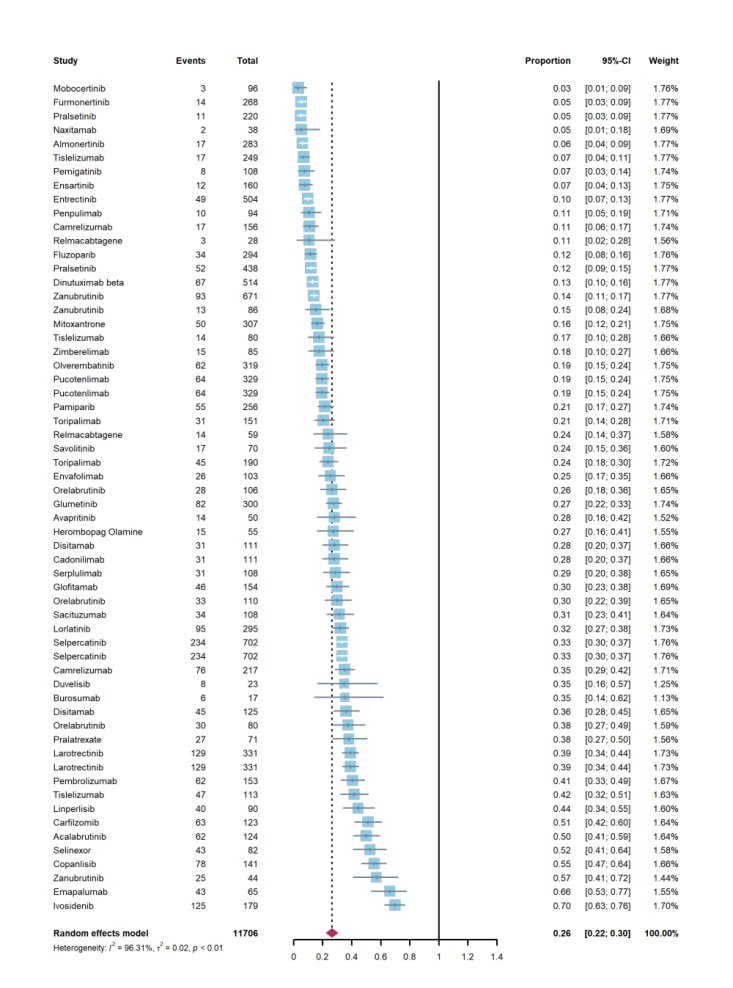


## Supplementary Figure 6.Forest plot of SAEs for single-arm pivotal trials for conditional approvals.

The block and whiskers represent the weighted average and 95% CI, respectively. The diamond represents the pooled estimates. SAEs are events that result in a patient's death, are life-threatening, result in hospitalisation or prolonged hospitalisation, and cause lasting or significant disability or dysfunction.


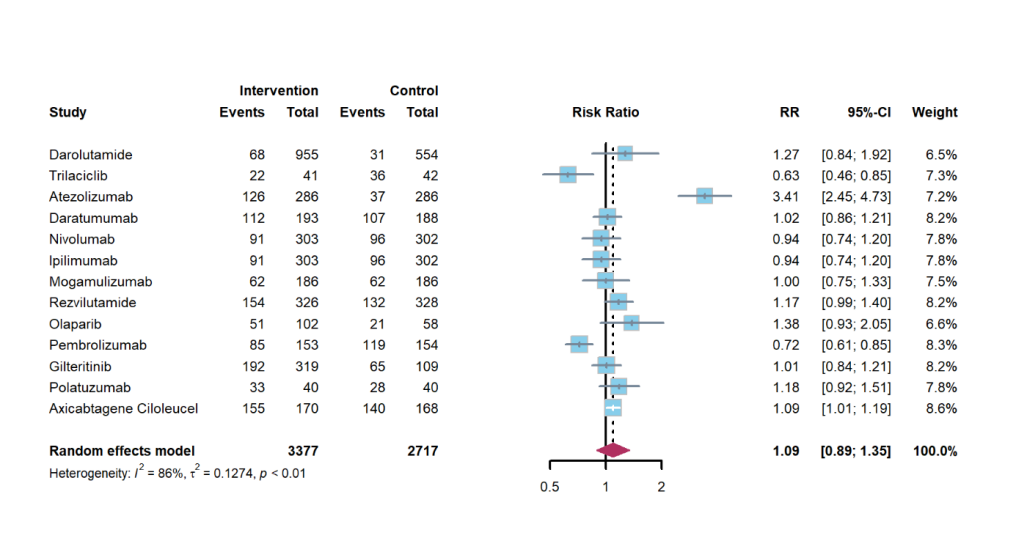


## Supplementary Figure 7.Forest plot of Grade ≥ 3 AEs for RCT pivotal trials for conditional approvals.

The block and whiskers represent the weighted average and 95% CI, respectively. The diamond represents the pooled estimates. The AEs level class according to the National Cancer Institute Common Terminology Criteria for AEs is as follows: Grade 1, Mild; asymptomatic or mild symptoms; clinical or diagnostic observations only; intervention not indicated. Grade 2, Moderate; minimal, local, or noninvasive intervention indicated; limiting age-appropriate instrumental activities of daily living (ADL). Grade 3, Severe or medically significant but not immediately life-threatening; hospitalization or prolongation of hospitalization indicated; disabling; limiting self-care ADL. Grade 4, Life-threatening consequences; urgent intervention indicated. Grade 5, Death related to AE.


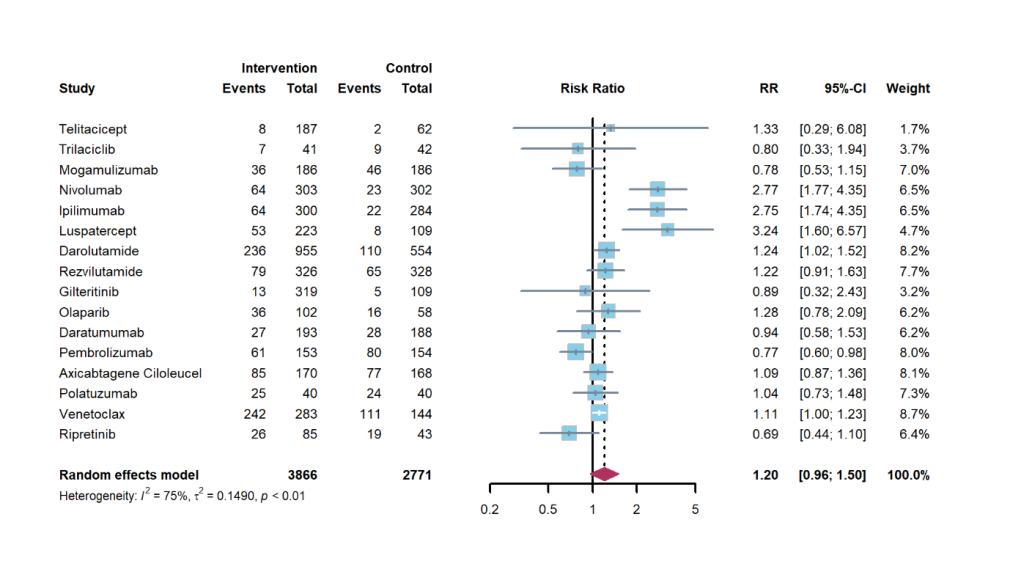


## Supplementary Figure 8.Forest plot of SAEs for RCT pivotal trials for conditional approvals.

The block and whiskers represent the weighted average and 95% CI **,** respectively. The diamond represents the pooled estimates.SAEs are events that result in a patient's death, are life-threatening, result in hospitalisation or prolonged hospitalisation, and cause lasting or significant disability or dysfunction.

# Supplementary Tables

## Supplementary Table 1.Comparison of four accelerated drug marketing registration procedures in China.

| **Accelerated Procedure** | **Applicable Criteria** | **Application stage** | **Benefits** |
| --- | --- | --- | --- |
| Conditional Approval (CA) | 1. Drugs for the treatment of serious life-threatening diseases for which there is no effective means of treatment ; 2. Drugs urgently needed for public health ; 3. Vaccines urgently needed for responding to major public health emergencies or other vaccines recognized as urgently needed by the National Health Commission | Communication during clinical trials and pre-market application, application in NDA. | 1)Accelerating marketing. Shorten the drug research and development process and accelerate the speed to market.  2)Allow early marketing based on surrogate endpoints, intermediate clinical endpoints or early clinical trial data. |
| Priority Review (PR) | 1) clinically urgent drugs in shortage, innovative and improved new drugs to prevent and treat major infectious diseases and rare diseases; 2) new varieties, dosage forms and specifications of medicines for children in line with the physiological characteristics of children; 3) vaccines and innovative vaccines needed for disease prevention and control; 4) drugs included in the breakthrough therapy drug procedure; 5) drugs in line with the conditional approval; 6) the NMPA stipulates that the other priority situations for review and approval. | Communicating and exchanging information and applying in tandem with the NDA. | 1)Reducing the review time of NDA ;  The review time was shortened from the standard 200 days to 130 days; the review time for overseas rare disease medicines with urgent clinical needs was shortened to 70 days .  2)After communication and confirmation, additional technical information can be submitted . |
| Breakthrough Therapy Designation (BTD) | 1)For serious or  life-threatening  Diseases ; 2)Preliminary clinical  evidence that suggests  substantial improvement  over existing therapies. | 1)Phase I and II clinical  Trials ;  2)Ideally, no later than  Phase III. | 1)Eligible for Priority  Review ;   1. Rolling submission ; 2. Intensive guidance from regulatory agencies. |
| Special Review and Approval (SRA) | In the emergence of public health emergency threats, as well as public health emergencies after the occurrence of public health emergencies, the NMPA can be decided by law on public health emergency prevention and treatment of drugs. | Before filing an application for registration. | 1) Shortening the timeframe. After the acceptance of the application for drug registration, the technical review of the registration declaration data will be carried out within 24 hours, and the on-site verification situation and relevant opinions will be reported to NMPA within 5 days. |

Abbreviations: NDA: New Drug Application; NMPA: National Medical Products Administration.

## Supplementary Table 2.Basic information on conditional approvals by the NMPA (2020-2023).

| **Number** | **Drug Name** | **Indication** | **Clinical development strategies** | **Address the unmet clinical needs** | **Drug type** | **Registration Categories** | **ATC Classification** | **Expedited program** | **Origin** | **Approval** Date |
| --- | --- | --- | --- | --- | --- | --- | --- | --- | --- | --- |
| 1 | Camrelizumab | Hepatocellular carcinoma | Local | No standard available treatments. | Therapeutic biologic product | 1 | Antineoplastics | PR+CA | Domestic | 3/5/2020 |
| 2 | Almonertinib | Non-small cell lung cancer | MRCT | Improved tolerance. | Chemical drug | 1 | Antineoplastics | PR+CA | Domestic | 3/17/2020 |
| 3 | Tislelizumab | Uroepithelial carcinoma | MRCT | No standard available treatments. | Therapeutic biologic product | 1 | Antineoplastics | PR+CA | Domestic | 4/9/2020 |
| 4 | Zanubrutinib | Mantle cell lymphoma  (MCL) | Local | No standard available treatments. | Chemical drug | 1 | Antineoplastics | PR+CA | Domestic | 6/2/2020 |
| 5 | Zanubrutinib | Chronic lymphocytic leukaemia (CLL)/small lymphocytic lymphoma (SLL) | Local | No standard available treatments. | Chemical drug | 1 | Antineoplastics | PR+CA | Domestic | 6/2/2020 |
| 6 | Pralatrexate | Peripheral T-cell lymphoma (PTCL) | Briging | No standard available treatments. | Chemical drug | 5.1 | Antineoplastics | PR+CA | Imported | 8/26/2020 |
| 7 | Ensartinib | Non-small cell lung cancer (NSCLC) | Local | Improved prognosis. | Chemical drug | 1 | Antineoplastics | PR+CA | Domestic | 11/17/2020 |
| 8 | Venetoclax | Acute Myeloid Leukaemia (AML) | MRCT | Improved prognosis. | Chemical drug | 5.1 | Antineoplastics | PR+CA | Imported | 12/2/2020 |
| 9 | Blinatumomab | Acute lymphoblastic leukaemia (AML) | Briging | No standard available treatments. | Therapeutic biologic product | 3.1 | Antineoplastics | PR+CA | Imported | 12/2/2020 |
| 10 | Fluzoparib | Cancer of the ovaries, fallopian tubes or primary peritoneal cancer | Local | Effectively mitigate the serious adverse effects associated with existing treatments. | Chemical drug | 1 | Antineoplastics | PR+CA | Domestic | 12/11/2020 |
| 11 | Orelabrutinib | Chronic lymphocytic leukaemia (CLL)/small lymphocytic lymphoma (SLL) | Local | Improved prognosis. | Chemical drug | 1 | Antineoplastics | PR+CA | Domestic | 12/25/2020 |
| 12 | Orelabrutinib | Massive cell lymphoma (MCL) | Local | No standard available treatments. | Chemical drug | 1 | Antineoplastics | PR+CA | Domestic | 12/25/2020 |
| 13 | Plerixafor | Multiple Myeloma(MM) | Waving | Effectively mitigate the serious adverse effects associated with existing treatments. | Chemical drug | 5.1 | Antineoplastics | CA | Imported | 8/26/2020 |
| 14 | Anlotinib | Medullary thyroid carcinoma | Local | First approved for marketing. | Chemical drug | 2.4 | Antineoplastics | PR+CA | Domestic | 1/30/2021 |
| 15 | Toripalimab | Nasopharyngeal Carcinoma | Local | No standard available treatments. | Therapeutic biologic product | 1 | Antineoplastics | PR+CA | Domestic | 2/10/2021 |
| 16 | Furmonertinib | Non-small cell lung cancer (NSCLC) | MRCT | Improved tolerance. | Chemical drug | 1 | Antineoplastics | PR+CA | Domestic | 3/3/2021 |
| 17 | Telitacicept | Systemic Lupus Erythematosus (SLE) | MRCT | Effectively mitigate the serious adverse effects associated with existing treatments. | Therapeutic biologic product | 1 | Immunomodulating agents | PR+CA | Domestic | 3/9/2021 |
| 18 | Pralsetinib | Non-small cell lung cancer (NSCLC) | Briging | Improved prognosis. | Chemical drug | 1 | Antineoplastics | PR+CA | Imported | 3/23/2021 |
| 19 | Avapritinib | Gastrointestinal mesenchymal tumour (GIST) | Briging | Improved prognosis. | Chemical drug | 5.1 | Antineoplastics | PR+CA | Imported | 3/30/2021 |
| 20 | Toripalimab | Uroepithelial carcinoma | Local | No standard available treatments. | Therapeutic biologic product | 1 | Antineoplastics | PR+CA | Domestic | 4/7/2021 |
| 21 | Camrelizumab | Nasopharyngeal Carcinoma | MRCT | No standard available treatments. | Therapeutic biologic product | 2.2 | Antineoplastics | PR+CA | Domestic | 4/27/2022 |
| 22 | Pamiparib | Cancer of the ovaries, fallopian tubes or primary peritoneal cancer | Local | Improved tolerance. | Chemical drug | 1 | Antineoplastics | PR+CA | Domestic | 4/30/2021 |
| 23 | Disitamab Vedotin | Stomach Cancer | Local | Existing treatments ineffective. | Therapeutic biologic product | 1 | Antineoplastics | PR+CA | Domestic | 6/8/2021 |
| 24 | Herombopag Olamine | Aplastic Anaemia (SAA) | Local | No standard available treatments. | Chemical drug | 1 | Blood and blood-forming organs | PR+CA | Domestic | 6/16/2021 |
| 25 | Zanubrutinib | Watson's Macroglobulinemia (WM) | Local | No standard available treatments. | Chemical drug | 2.4 | Antineoplastics | PR+CA | Domestic | 6/21/2021 |
| 26 | Savolitinib | Non-small cell lung cancer | Local | First approved for marketing. | Chemical drug | 1 | Antineoplastics | PR+CA | Domestic | 6/22/2021 |
| 27 | Tislelizumab | Hepatocellular carcinoma (HCC) | MRCT | Existing treatments ineffective. | Therapeutic biologic product | 1 | Antineoplastics | CA | Domestic | 6/22/2021 |
| 28 | Carfilzomib | Multiple myeloma | Briging | Existing treatments ineffective. | Chemical drug | 5.1 | Antineoplastics | CA | Imported | 7/6/2021 |
| 29 | Azvudine | HIV-1 infection | Local | Improved tolerance. | Chemical drug | 1 | Anti-infectives for systemic use | PR+CA | Domestic | 7/20/2021 |
| 30 | Penpulimab | Hodgkin's lymphoma | Local | No standard available treatments. | Therapeutic biologic product | 1 | Antineoplastics | CA | Domestic | 8/3/2021 |
| 31 | Zimberelimab | Hodgkin's lymphoma | Local | No standard available treatments. | Therapeutic biologic product | 1 | Antineoplastics | CA | Domestic | 8/25/2021 |
| 32 | Relmacabtagene | B-cell lymphoma | Local | Existing treatments ineffective. | Therapeutic biologic product | 1 | Antineoplastics | PR+CA | Domestic | 9/1/2021 |
| 33 | Daratumumab | Primary light chain amyloidosis | MRCT | First approved for marketing. | Therapeutic biologic product | 2.2 | Antineoplastics | PR+CA | Imported | 9/30/2021 |
| 34 | Orebatinib | Chronic myeloid leukaemia | Local | Improved tolerance. | Chemical drug | 1 | Antineoplastics | PR+CA | Domestic | 11/24/2021 |
| 35 | Envafolimab | Highly indolent (MSI-H) or mismatch repair gene-deficient (dMMR) advanced solid tumours | Local | Improved prognosis. | Therapeutic biologic product | 1 | Antineoplastics | PR+CA | Domestic | 11/24/2021 |
| 36 | Selinexor | Multiple myeloma | Briging | Improved tolerance. | Chemical drug | 5.1 | Antineoplastics | PR+CA | Imported | 12/14/2021 |
| 37 | Disitamab Vedotin | Uroepithelial carcinoma | Local | No standard available treatments. | Therapeutic biologic product | 2.2 | Antineoplastics | BTD+PR+CA | Domestic | 12/31/2021 |
| 38 | Atezolizumab | Non-small cell lung cance | MRCT | Improved tolerance. | Therapeutic biologic product | 3.1 | Antineoplastics | PR+CA | Imported | 4/29/2021 |
| 39 | Olaparib | Prostate cancer | Waving | No standard available treatments. | Chemical drug | 5.1 | Antineoplastics | PR+CA | Imported | 6/16/2021 |
| 40 | Burosumab | X -linked Hypopho- sphatemic Rickets( XLH) | Waving | Improved tolerance. | Therapeutic biologic product | 3.1 | Musculoskeletal system | PR+CA | Imported | 1/18/2021 |
| 41 | Burosumab | Tumor-induced Osteomalacia(TIO) | Waving | Improved tolerance. | Therapeutic biologic product | 3.1 | Musculoskeletal system | PR+CA | Imported | 3/25/2021 |
| 42 | Darolutamide | Prostate cancer | Waving | No standard available treatments. | Chemical drug | 5.1 | Antineoplastics | PR+CA | Imported | 2/2/2021 |
| 43 | Dinutuximab beta | Neuroblastoma | Waving | No standard available treatments. | Therapeutic biologic product | 3.1 | Antineoplastics | PR+CA | Imported | 8/12/2021 |
| 44 | Gilteritinib fumarate | Leukaemia | Waving | No standard available treatments. | Chemical drug | 5.1 | Antineoplastics | PR+CA | Imported | 1/30/2021 |
| 45 | Nivolumab | M alignant P leural M esothelioma | MRCT | Improved prognosis. | Therapeutic biologic product | 3.1 | Antineoplastics | PR+CA | Imported | 6/8/2021 |
| 46 | Pembrolizumab | Colorectal cancer | Waving | No standard available treatments. | Therapeutic biologic product | 3.1 | Antineoplastics | PR+CA | Imported | 6/8/2021 |
| 47 | Ripretinib | Gastrointestinal stromal tumors | Briging | Improved prognosis. | Chemical drug | 5.1 | Antineoplastics | PR+CA | Imported | 3/30/2021 |
| 48 | Mitoxantrone liposomal | Peripheral T-cell lymphoma | Local | No standard available treatments. | Chemical drug | 2.2 | Antineoplastics | PR+CA | Domestic | 1/7/2022 |
| 49 | Ipilimumab | M alignant P leural M esothelioma | MRCT | Improved prognosis. | Therapeutic biologic product | 3.1 | Antineoplastics | PR+CA | Imported | 6/8/2021 |
| 50 | Icaritin | Liver Cancer | Local | Effectively mitigate the serious adverse effects associated with existing treatments. | Traditional Chinese medicine | 1.2 | Antineoplastics | PR+CA | Domestic | 1/10/2022 |
| 51 | Linperlisib | follicular lymphoma | Local | No standard available treatments. | Chemical drug | 1 | Antineoplastics | BTD+PR+CA | Domestic | 11/8/2022 |
| 52 | Pyrrolitinib | Breast cancer | Local | Improved tolerance. | Chemical drug | 2.4 | Antineoplastics | PR+CA | Domestic | 5/31/2022 |
| 53 | Rezvilutamide | Prostate adenocarcinoma | MRCT | Improved prognosis. | Chemical drug | 1 | Antineoplastics | BTD+PR+CA | Domestic | 6/28/2022 |
| 54 | Serplulimab | Advanced malignance | Local | First approved for marketing. | Therapeutic biologic product | 1 | Antineoplastics | PR+CA | Domestic | 3/29/2022 |
| 55 | Tislelizumab | Advanced malignance | MRCT | Improved prognosis | Therapeutic biologic product | 2.2 | Antineoplastics | PR+CA | Domestic | 3/8/2022 |
| 56 | Pucotenlimab | Melanoma | Local | Improved prognosis. | Therapeutic biologic product | 1 | Antineoplastics | CA | Domestic | 9/20/2022 |
| 57 | Cadonilimab | Cervical Cancer | Local | First approved for marketing. | Therapeutic biologic product | 1 | Antineoplastics | BTD+PR+CA | Domestic | 6/28/2022 |
| 58 | Pucotenlimab | Advanced malignance | Local | First approved for marketing. | Therapeutic biologic product | 1 | Antineoplastics | PR+CA | Domestic | 7/19/2022 |
| 59 | Relmacabtagene | follicular lymphoma | Local | No standard available treatments. | Therapeutic biologic product | 2.2 | Antineoplastics | BTD+PR+CA | Domestic | 9/30/2022 |
| 60 | Pralsetinib | Thyroid Cancer | Briging | First approved for marketing. | Chemical drug | 5.1 | Antineoplastics | BTD+PR+CA | Imported | 3/8/2022 |
| 61 | Duvelisib | follicular lymphoma | Briging | Improved tolerance. | Chemical drug | 5.1 | Antineoplastics | PR+CA | Imported | 3/16/2022 |
| 62 | Larotrectinib | Advanced malignance | MRCT | No standard available treatments. | Chemical drug | 5.1 | Antineoplastics | PR+CA | Imported | 6/23/2022 |
| 63 | Larotrectinib | Advanced malignance | MRCT | No standard available treatments. | Chemical drug | 5.1 | Antineoplastics | PR+CA | Imported | 4/8/2022 |
| 64 | Mobocertinib | Non-Small Cell Lung Cancer | MRCT | First approved for marketing. | Chemical drug | 1 | Antineoplastics | BTD+PR+CA | Imported | 1/10/2023 |
| 65 | Pemigatinib | Bile Duct Cancer | Briging | No standard available treatments. | Chemical drug | 5.1 | Antineoplastics | PR+CA | Imported | 3/29/2022 |
| 66 | Lorlatinib | Non-Small Cell Lung Cancer | Local | Improved prognosis. | Chemical drug | 5.1 | Antineoplastics | PR+CA | Imported | 4/27/2022 |
| 67 | Ivosidenib | Acute Myeloid Leukemia | Briging | Improved prognosis. | Chemical drug | 5.1 | Antineoplastics | PR+CA | Imported | 1/30/2022 |
| 68 | Trametinib | Non-Small Cell Lung Cancer | Briging | First approved for marketing. | Chemical drug | 5.1 | Antineoplastics | PR+CA | Imported | 3/22/2022 |
| 69 | Dabrafenib | Non-Small Cell Lung Cancer | Briging | First approved for marketing. | Chemical drug | 5.1 | Antineoplastics | PR+CA | Imported | 3/22/2022 |
| 70 | Entrectinib | Advanced malignance | MRCT | First approved for marketing. | Chemical drug | 5.1 | Antineoplastics | PR+CA | Imported | 7/26/2022 |
| 71 | Selpercatinib | Medullary thyroid carcinoma | Briging | First approved for marketing. | Chemical drug | 5.1 | Antineoplastics | PR+CA | Imported | 9/30/2022 |
| 72 | Selpercatinib | Non-Small Cell Lung Cancer | Briging | First approved for marketing. | Chemical drug | 5.1 | Antineoplastics | PR+CA | Imported | 9/30/2022 |
| 73 | Trilaciclib | Small Cell Lung Cancer | Briging | No standard available treatments. | Chemical drug | 5.1 | Antineoplastics | PR+CA | Imported | 7/12/2022 |
| 74 | Emapalumab | hemophagocytic lymphohistiocytosis | Waving | First approved for marketing. | Therapeutic biologic product | 3.1 | Antineoplastics | PR+CA | Imported | 3/8/2022 |
| 75 | Luspatercept | β-mediterranean anemia | Waving | Improved prognosis. | Therapeutic biologic product | 3.1 | Blood and blood-forming organs | PR+CA | Imported | 1/25/2022 |
| 76 | Blinatumomab | Leukaemia | Waving | No standard available treatments. | Therapeutic biologic product | 3.1 | Antineoplastics | PR+CA | Imported | 4/27/2022 |
| 77 | Trodelvy | Breast cancer | Briging | No standard available treatments. | Therapeutic biologic product | 3.1 | Antineoplastics | PR+CA | Imported | 6/7/2022 |
| 78 | Mogamulizumab | Mycosis fungoides | Waving | Existing treatments ineffective. | Therapeutic biologic product | 3.1 | Antineoplastics | PR+CA | Imported | 10/11/2022 |
| 79 | Naxitamab | Neuroblastoma | MRCT | Improved prognosis. | Therapeutic biologic product | 3.1 | Antineoplastics | PR+CA | Imported | 11/30/2022 |
| 80 | Polatuzumab Vedotin | B lymphocytic lymphoma | Briging | No standard available treatments. | Therapeutic biologic product | 3.1 | Antineoplastics | PR+CA | Imported | 1/10/2023 |
| 81 | Glumetinib | Small Cell Lung Cancer | MRCT | Existing treatments ineffective. | Chemical drug | 1 | Antineoplastics | BTD+PR+CA | Domestic | 3/7/2023 |
| 82 | Sunvozertinib | Non-Small Cell Lung Cancer | Local | Improved tolerance. | Chemical drug | 1 | Antineoplastics | BTD+PR+CA | Domestic | 8/24/2023 |
| 83 | Glofitamab | B lymphocytic lymphoma | MRCT | No standard available treatments. | Therapeutic biologic product | 1 | Antineoplastics | BTD+PR+CA | Imported | 11/7/2023 |
| 84 | Bozitinib | Non-Small Cell Lung Cancer | MRCT | First approved for marketing. | Chemical drug | 1 | Antineoplastics | BTD+PR+CA | Domestic | 11/17/2023 |
| 85 | Equecabtagene Autoleucel | Multiple myeloma | MRCT | First approved for marketing. | Therapeutic biologic product | 1 | Antineoplastics | BTD+PR+CA | Domestic | 6/30/2023 |
| 86 | Inaticabtagene Autoleucel | B lymphocytic lymphoma | MRCT | First approved for marketing. | Therapeutic biologic product | 1 | Antineoplastics | BTD+PR+CA | Domestic | 11/7/2023 |
| 87 | Naloxumab | Giant cell tumor | MRCT | First approved for marketing. | Therapeutic biologic product | 1 | Antineoplastics | PR+CA | Domestic | 9/5/2023 |
| 88 | Centella Asiatica extract | Diabetic foot ulcer | MRCT | First approved for marketing. | Traditional Chinese medicine | 1.1 | Dermatological | CA | Domestic | 11/9/2023 |
| 89 | Acalabrutinib | Massive cell lymphoma (MCL) | Briging | No standard available treatments. | Chemical drug | 5.1 | Antineoplastics | CA | Imported | 2/21/2023 |
| 90 | Socazolimab | Cervical Cancer | MRCT | Existing treatments ineffective. | Therapeutic biologic product | 1 | Antineoplastics | BTD+CA | Domestic | 12/19/2023 |
| 91 | Copanlisib | follicular lymphoma | Briging | Existing treatments ineffective. | Chemical drug | 5.1 | Antineoplastics | PR+CA | Imported | 5/19/2023 |
| 92 | Axicabtagene Ciloleucel | Large B cell lymphoma | MRCT | Improved prognosis. | Therapeutic biologic product | 3.2 | Antineoplastics | PR+CA | Domestic | 6/21/2023 |
| 93 | Orelabrutinib | Marginal zone lymphomas（MZL） | Local | Existing treatments ineffective. | Chemical drug | 2.4 | Antineoplastics | PR+CA | Domestic | 417/2023 |
| 94 | Zimberelimab | Cervical Cancer | MRCT | Existing treatments ineffective. | Therapeutic biologic product | 2.2 | Antineoplastics | CA | Domestic | 6/30/2023 |
| 95 | Sugemalimab | Extranodal NK-T-Cell Lymphoma | Local | First approved for marketing. | Therapeutic biologic product | 2.2 | Antineoplastics | BTD+PR+CA | Domestic | 10/27/2023 |
| 96 | Amubarvimab | Treatment of COVID-19 | / | Drugs urgently needed for public health | Therapeutic biologic product | 1 | Anti-infectives for systemic use | CA+SRA | Domestic | 2021 |
| 97 | Romlusevimab | Treatment of COVID-19 | / | Drugs urgently needed for public health | Therapeutic biologic product | 1 | Anti-infectives for systemic use | CA+SRA | Domestic | 2021 |
| 98 | Azvudine | Treatment of COVID-19 | / | Drugs urgently needed for public health | Chemical drug | 2.4 | Anti-infectives for systemic use | CA+SRA | Domestic | 2022 |
| 99 | Nirmatrelvir Tablets/Ritonavir Tablets;(co-packaged) | For the treatment of adult patients with mild to moderate COVID-19 associated with high risk factors for progression to severe disease. | / | Drugs urgently needed for public health | Chemical drug | 5.1 | Anti-infectives for systemic use | CA+SRA | Imported | 2022 |
| 100 | Molnupiravir | For the treatment of adult patients with mild to moderate COVID-19 with high risk factors for progression to severe disease | / | Drugs urgently needed for public health | Chemical drug | 5.1 | Anti-infectives for systemic use | CA+SRA | Imported | 2022 |
| 101 | Deuremidevir Hydrobromide | For the treatment of adult patients with mild-to-moderate COVID-19 | / | Drugs urgently needed for public health | Chemical drug | 1 | Anti-infectives for systemic use | CA+SRA | Domestic | 1/28/2023 |
| 102 | Simnotrelvir Tablets/Ritonavir Tablets(co-packaged) | For the treatment of adult patients with mild-to-moderate COVID-19 | / | Drugs urgently needed for public health | Chemical drug | 1 | Anti-infectives for systemic use | CA+SRA | Domestic | 1/28/2023 |
| 103 | Leritrelvir | For the treatment of adult patients with mild-to-moderate COVID-19 | / | Drugs urgently needed for public health | Chemical drug | 1 | Anti-infectives for systemic use | CA+SRA | Domestic | 2023 |

Abbreviations: NMPA: National Medical Products Administration; CA: Conditional Approval; BTD: Breakthrough Therapy Designation; PR: Priority Review; SRA: Special Review and Approval.

## Supplementary Table S3. The definition of drug registration classification.

| Drug types | Registration Categories | Specific definitions |
| --- | --- | --- |
| Chemical drugs | Class 1 | Innovative new drugs that have never been marketed globally |
|  | Class 2.2 | New dosage forms (including new delivery systems), new prescription processes, and new routes of administration that contain known active ingredients and that have clear clinical advantages |
|  | Class 2.4 | With new indications for approved compounds |
|  | Class 5.1 | Original or improved new drugs that have received marketing approval overseas. |
| Therapeutic biological products | Class 1 | Innovative therapeutic biological products that have not been marketed globally |
|  | Class 2.2 | Addition of new indications that are not approved either domestically or internationally and/or changes in the dosing population |
|  | Class 3.1 | Application of therapeutic biological products produced outside China that have been marketed overseas and have not been marketed in China |
|  | Class 3.2 | Therapeutic biological products that have been marketed abroad and not in China are declared to be produced and marketed in China |
| Traditional Chinese medicines | Class 1.1 | Traditional Chinese medicines compound preparation |
|  | Class 1.2 | Extracts and preparations obtained from single substances, including plants, animals, minerals, etc |

## Supplementary Table S4. The characteristics of pivotal clinical trials.

| **Number** | Drug Name | Indications | Trial Number | **Trial Design** | Enrollment, No. | Study endpoints |
| --- | --- | --- | --- | --- | --- | --- |
| 1 | Zanubrutinib | Mantle cell lymphoma  (MCL) | CTR20160888 | Non-randomised，open, single-arm phase II clinical study | 86 | RR |
| 2 | Zanubrutinib | Leukaemia | CTR20170208 | Non-randomised，open, single-arm phase II clinical study | 91 | RR |
| 3 | Ensartinib | Non-small cell lung cancer (NSCLC) | CTR20170762 | Non-randomised, open, single-arm phase II clinical study | 160 | RR |
| 4 | Almonertinib | Non-small cell lung cancer | NCT02981108 | International multicentre, non-randomised, open, single-arm phase II clinical study | 364 | RR |
| 5 | Fluzoparib | Ovarian cancer | CTR20180156 | Non-randomised, open, single-arm phase Ib clinical study | 113 | RR |
| 6 | Orelabrutinib | Chronic lymphocytic leukaemia (CLL)/small lymphocytic lymphoma (SLL) | CTR20180263 | Non-randomised, open, single-arm phase I/II clinical study | 80 | RR |
| 7 | Orelabrutinib | Massive cell lymphoma (MCL) | CTR20180196 | Non-randomised, open, single-arm phase I/II clinical study | 106 | RR |
| 8 | Camrelizumab | Liver Cancer | CTR20160871 | Randomised, open, single-arm phase II/Ⅲ clinical study | 220 | RR |
| 9 | Tislelizumab | Uroepithelial carcinoma | CTR20170071 | International multicentre, non-randomised, open, single-arm phase II clinical study | 221 | RR |
| 10 | Pralatrexate | Peripheral T-cell lymphoma (PTCL) | CTR20150402 | Non-randomised, open, single-arm phase III clinical study | 72 | RR |
|  |  |  | NCT00364923 | International multicentre, non-randomised, open, single-arm phase II clinical study | 111 | RR |
| 11 | Venetoclax | Acute Myeloid Leukaemia (AML) | CTR20171498 | International multicentre, randomised, double-blind, placebo-controlled phase III clinical study | 490 | OS |
|  |  |  | NCT02203773 | International multicentre, non-randomised, open, single-arm phase Ib clinical study | 67 | RR |
| 12 | Plerixafro | Multiple Myeloma | NCT00741780 | International multicentre, randomised, double-blind, placebo-controlled phase III clinical study | 164 | Proportion of patients with ≥6×10^6^ CD34+ cells/kg collected from peripheral blood on 2 or fewer collection days |
| 13 | Blinatumomab | Acute lymphoblastic leukaemia | NCT01471782 | International multicentre, non-randomised, open, single-arm phase II clinical study | 405 | RR |
|  |  |  | CTR20170176 | Non-randomised, open, single-arm phase Ⅲ clinical study | 67 | RR |
| 14 | Avapritinib | Gastrointestinal mesenchymal tumour (GIST) | NCT02508532 | International multicentre, non-randomised, open, single-arm phase I clinical study | 62 | RR |
|  |  |  | CTR20191409 | Non-randomised, open, single-arm phase I/II clinical study | 50 | RR |
| 15 | Atezolizumab | Non-Small Cell Lung Cancer | NCT02409342 | International multicentre, randomised, open, positive drug-controlled phase III clinical study | 572 | OS |
| 16 | Azvudine | HIV-1 infection | CTR20171345 | Randomised, double-blind, positive drug-controlled phase II clinical study | 172 | RNA <50copies/ml % of subjects |
| 17 | Olaparib | Prostate cancer | PROfound | International multicentre, randomised, open, positive drug-controlled phase III clinical study | 347 | PFS |
| 18 | Orelabrutinib | Leukaemia | CTR20190274 | Non-randomised, open, single-arm phase II clinical study | 41 | RR |
| 19 | Burosumab | X-linked hypophosphatemia（XLH） | NCT02915705 | International multicentre, randomised, open, positive drug-controlled phase III clinical study | 61 | Radiographic Global Impression of Change (RGI-C) Global Score at Week 40 |
| 20 | Burosumab | Tumor-induced osteomalacia（TIO） | NCT02304367 | International multicentre,  non- randomised, open, single-arm phase II clinical study | 17 | Radiographic Global Impression of Change (RGI-C) Global Score at Week 40 |
| 21 | Daratumumab | Multiple Myeloma | CTR20182174 | International multicentre,  randomised, open, positive drug-controlled phase III clinical study | 393 | OS |
| 22 | Darolutamide | Prostate cancer | NCT02200614 | International multicentre, randomised, double-blind, placebo-controlled phase III clinical study | 1509 | PFS |
| 23 | Dinutuximab beta | Neuroblastoma | NA | International multicentre, non-randomised, open, single-arm phase I/Ⅱ clinical study | 379 | RR |
| 24 | Envafolimab | Highly indolent (MSI-H) or mismatch repair gene-deficient (dMMR) advanced solid tumours | CTR20181127 | Non-randomised, open, single-arm phase II clinical study | 103 | RR |
| 25 | Gilteritinib | Leukaemia | NCT02421939 | International multicentre, randomised, open,positive drug-controlled phase III clinical study | 371 | OS |
| 26 | Herombopag Olamine | Severe aplastic anemia | CTR20180868 | Single-arm, non-randomised, open，phase I/II clinical trial | 55 | HR (⩾1 lineage; 18-week) |
| 27 | Furmonertinib | Non-small cell lung cancer (NSCLC) | NCT02981108 | International multicentre, non-randomised, open, single-arm phase II clinical study | 364 | RR |
| 28 | Nivolumab | M alignant P leural M esothelioma | CTR20170913 | International multicentre, randomised, open, positive drug-controlled ,phase III clinical study | 610 | OS |
| 29 | Pembrolizumab | Colorectal cancer | NCT02563002 | International multicentre, randomised, open, positive drug-controlled ,phase III clinical study | 307 | PFS |
| 30 | Pamiparib | Ovarian cancer | CTR20160828 | Non-randomised, open, single-arm phase I/II clinical study | 128 | RR |
| 31 | Penpulimab | Hodgkin's lymphoma | CTR20181311 | Non-randomised, open, single-arm phase I/II clinical study | 94 | RR |
| 32 | Pralsetinib | Non-small cell lung cancer (NSCLC) | CTR20190767 | International multicentre, non-randomised, open, single-arm phase I/II clinical study | 698 | RR |
| 33 | Relmacabtagene autoleucel | Large B-cell lymphoma | CTR20182325 | Randomised, open, phase II clinical study | 72 | RR |
| 34 | Ripretinib | Gastrointestinal stromal tumors | NCT03353753 | International multicentre, randomised, double-blind, placebo-controlled phase III clinical study | 129 | PFS |
|  |  |  | CTR20192728 | Non-randomised, open, single-arm phase II clinical study | 39 | PFS |
| 35 | Selinexor | Multiple myeloma | NCT02336815 | International multicentre, non-randomised, open, single-arm phase II clinical study | 202 | RR |
|  |  |  | CTR20190858 | Non-randomised, open, single-arm phase II clinical study | 82 | RR |
| 36 | Zimberelimab | Hodgkin's lymphoma | CTR20181224 | Non-randomised, open, single-arm phase II clinical study | 85 | RR |
| 37 | Savolitinib | Non-small cell lung cancer | CTR20160581 | Non-randomised, open, single-arm phase II clinical study | 76 | RR |
| 38 | Toripalimab | Nasopharyngeal Carcinoma | CTR20160740 | Open,non-randomised, single-arm phase Ib/II clinical study | 401 | RR |
| 39 | Toripalimab | Uroepithelial carcinoma | CTR20170347 | Non-randomised, open, single-arm phase II clinical study | 172 | RR |
| 40 | Tislelizumab | Liver Cancer | CTR20171257 | International multicentre, non-randomised, open, single-arm phase II clinical study | 249 | RR |
| 41 | Anlctinib | Medullary thyroid carcinoma | NCT02586350 | International multicentre, randomised, double-blind, placebo-controlled phase Ⅱ clinical study | 91 | PFS |
| 42 | Mitoxantrone liposomal | peripheral T-cell lymphoma | CTR20182224 | Non-randomised, open, single-arm phase II clinical study | 108 | RR |
| 43 | Ipilimumab | M alignant P leural M esothelioma | CTR20170913 | International multicentre, randomised, open, positive drug-controlled ,phase III clinical study | 610 | OS |
| 44 | Icaritin | Liver Cancer | CTR20170667 | Randomised, double-blind, positive drug-controlled phase III clinical study | 284 | OS |
| 45 | Zanubrutinib | Watson's Macroglobulinemia (WM) | CTR20170208 | Non-randomised, open, single-arm phase II clinical study | 44 | RR |
| 46 | Carfilzomib | Multiple myeloma | CTR20160857 | Non-randomised, open, single-arm phase III clinical study | 126 | RR |
| 47 | Camrelizumab | Nasopharyngeal Carcinoma | NCT03558191 | International multicentre, non-randomised, open, single-arm phase II clinical study | 156 | RR |
| 48 | Telitacicept | Systemic Lupus Erythematosus (SLE) | NCT02885610 | International multicentre, randomised, double-blind, placebo-controlled phase II clinical study | 249 | Week 48 SLE Response Index 4 (SRI4) response rate |
| 49 | Disitamab Vedotin | Stomach Cancer | CTR20180844 | Non-randomised, open, single-arm phase II clinical study | 127 | RR |
| 50 | Disitamab Vedotin | Uroepithelial carcinoma | CTR20180438 | Non-randomised, open, single-arm phase II clinical study | 107 | RR |
| 51 | Linperlisib | follicular lymphoma | CTR20182094 | Non-randomised, open, single-arm phase II clinical study | 93 | RR |
| 52 | Pyrrolitinib | Breast cancer | CTR20180941 | Randomised, double-blind, placebo-controlled phase III clinical study | 355 | tpCR |
| 53 | Rezvilutamide | Prostate adenocarcinoma | CTR20180560 | Randomised, open, positive drug-controlled,phase III clinical study | 197 | PFS |
| 54 | Serplulimab | Advanced malignance | CTR20190719 | Open, non-randomised,single-arm phase II clinical study | 108 | RR |
| 55 | Tislelizumab | Advanced malignance | CTR20180867 | Open, non-randomised,single-arm phase II clinical study | 84 | RR |
| 56 | Pucotenlimab | Melanoma | CTR20181269 | Open, non-randomised,single-arm phase II clinical study | 158 | RR |
| 57 | Cadonilimab | Cervical Cancer | CTR20182027 | Non-randomised, open, single-arm phase Ib/II clinical study | 338 | RR |
| 58 | Pucotenlimab | Advanced malignance | CTR20181881 | Open, non-randomised,single-arm phase II clinical study | 119 | RR |
| 59 | Relmacabtagene autoleucel | follicular lymphoma | CTR20182325 | Non-randomised, open, single-arm phase I/II clinical study | 72 | RR |
| 60 | Pralsetinib | Thyroid Cancer | CTR20190767 | International multicentre, non-randomised, open, single-arm phase I/II clinical study | 87 | RR |
|  |  |  | CTR20190767 | International multicentre, non-randomised, open, single-arm phase I/II clinical study | 32 | RR |
| 61 | Duvelisib | Follicular Lymphoma | NCT01882803 | International multicentre, open,non-randomised, single-arm phase II clinical study | 129 | RR |
|  |  |  | CTR20200132 | Open, non-randomised,single-arm phase II clinical study | 23 | RR |
| 62 | Larotrectinib | Advanced malignance | CTR20190625 | International multicentre, randomised, open, single-arm phase II clinical study | 259 | RR |
| 63 | Larotrectinib | Advanced malignance | CTR20190625 | International multicentre, randomised, open, single-arm phase II clinical study | 259 | RR |
| 64 | Mobocertinib | Non-Small Cell Lung Cancer | CTR20190582 | International multicentre, open,non-randomised, single-arm phase II clinical study | 107 | RR |
| 65 | Pemigatinib | Bile Duct Cancer | NCT02924376 | International multicentre, open,non-randomised, single-arm phase II clinical study | 147 | RR |
|  |  |  | CTR20192515 | Open, non-randomised,single-arm phase II clinical study | 30 | RR |
| 66 | Lorlatinib | Non-Small Cell Lung Cancer | CTR20181867 | Open,non-randomised, single-arm phase II clinical study | 109 | RR |
| 67 | Ivosidenib | Acute Myeloid Leukemia | NCT02074839 | International multicentre, open, non-randomised, single-arm phase I clinical study | 291 | RR |
|  |  |  | CTR20192088 | Open,non-randomised, single-arm phase I clinical study | 30 | RR |
| 68 | Trametinib | Non-Small Cell Lung Cancer | NCT01336634 | International multicentre, open,non-randomised, single-arm phase II clinical study | 177 | RR |
|  |  |  | CTR20200259 | Open,non-randomised, single-arm phase II clinical study | 20 | RR |
| 69 | Dabrafenib | Non-Small Cell Lung Cancer | NCT01336634 | International multicentre, open,non-randomised, single-arm phase II clinical study | 177 | RR |
|  |  |  | CTR20200259 | Open,non-randomised, single-arm phase II clinical study | 20 | RR |
| 70 | Entrectinib | Advanced malignance | NCT02568267 | International multicentre, open,non-randomised, single-arm phase II clinical study | 74 | RR |
| 71 | Selpercatinib | Medullary thyroid carcinoma | NCT03157128 | International multicentre, non-randomised, open, single-arm phase I/II clinical study | 55 | RR |
|  |  |  | NCT03157128 | International multicentre, non-randomised, open, single-arm phase I/II clinical study | 88 | RR |
|  |  |  | LIBRETTO-321 | Open,non-randomised, single-arm phase II clinical study | 26 | RR |
| 72 | Selpercatinib | Non-Small Cell Lung Cancer | NCT03157128 | International multicentre, non-randomised, open, single-arm phase I/II clinical study | 105 | RR |
|  |  |  | NCT03157128 | International multicentre, non-randomised, open, single-arm phase I/II clinical study | 39 | RR |
|  |  |  | LIBRETTO-321 | Open,non-randomised, single-arm phase II clinical study | 26 | RR |
| 73 | Trilaciclib | Small Cell Lung Cancer | NCT02499770 | International multicentre, randomised,double-blind, placebo-controlled phase I/II clinical study | 75 | DSN |
|  |  |  | CTR20210562 | Randomised,double-blind, placebo-controlled phase Ⅲ clinical study | 83 | DSN |
| 74 | Emapalumab | Hemophagocytic lymphohistiocytosis | NCT02069899 | International multicentre, non-randomised, open, single-arm phase II/III clinical study | 58 | RR |
| 75 | Luspatercept | β-mediterranean anemia | NCT02604433 | International multicentre, randomised, double-blind, placebo-controlled, phase III clinical study | 336 | Percentage of Participants Who Achieved Erythroid Response |
| 76 | Blinatumomab | Leukaemia | NCT02000427 | International multicentre, non-randomised, open, single-arm phase II clinical study | 45 | RR |
| 77 | Sacituzumab | Breast cancer | NCT01631552 | International multicentre, open, non-randomised,single-arm, phase II clinical study | 108 | RR |
|  |  |  | NCT04454437 | Open,non-randomised, single-arm phase II clinical study | 80 | RR |
| 78 | Mogamulizumab | Mycosis fungoides | NCT01728805 | International multicentre, randomised, open, positive-controlled phase III clinical study | 372 | PFS |
| 79 | Naxitamab | Neuroblastoma | NCT03363373 | International multicentre, open, non-randomised,single-arm phase II clinical study | 22 | RR |
|  |  |  | NCT01757626 | Open,non-randomised, single-arm phase II clinical study | 38 | RR |
| 80 | Polatuzumab Vedotin | B lymphocytic lymphoma | G029365 | International multicentre, randomised, open, positive-controlled phase I/II clinical study | 80 | OS |
|  |  |  | CTR20192186 | Randomised,double-blind, placebo-controlled, phase III clinical study | 42 | RR |
| 81 | Glumetinib | Small Cell Lung Cancer | CTR20190900 | International multicentre, open, non-randomised,single-arm phaseⅠ/II clinical study | 345 | RR |
| 82 | Sunvozertinib | Non-Small Cell Lung Cancer | CTR20211009 | Non-randomised, open, single-arm phase II clinical study | 104 | RR |
| 83 | Glofitamab | B lymphocytic lymphoma | NCT03075696 | International multicentre, non-randomised, open, single-arm phase I/II clinical study | 132 | RR |
| 84 | Vebreltinib | Non-Small Cell Lung Cancer | NCT04258033 | International multicentre, open, non-randomised,single-arm phase II clinical study | 52 | RR |
| 85 | Equecabtagene Autoleucel | Multiple myeloma | NCT05066646 | International multicentre, non-randomised, open, single-arm phase I/II clinical study | 107 | RR |
| 86 | Inaticabtagene Autoleucel | B lymphocytic lymphoma | NCT04684147 | International multicentre, non-randomised, open, single-arm ,phase II clinical study | 39 | RR |
| 87 | Naloxumab | Giant cell tumor | NCT04255576 | International multicentre, non-randomised, open, single-arm phase Ib/II clinical study | 139 | RR |
| 88 | Centella Asiatica extract | diabetic foot ulcer | NCT01898923 | International multicentre, randomised, double-blind, placebo-controlled, phase III clinical study | 236 | Healing Rate |
| 89 | Acalabrutinib | Massive cell lymphoma (MCL) | NCT02213926 | International multicentre, randomised, open, single-arm phase II clinical study | 124 | RR |
|  |  |  | CTR20181987 | Non-randomised, open, single-arm phase I/II clinical study | 34 | RR |
| 90 | Socazolimab | Cervical Cancer | NA | International multicentre, randomised, open, single-arm phase I clinical study | 104 | RR |
| 91 | Copanlisib | follicular lymphoma | NCT01660451 | International multicentre, randomised, open, single-arm phase II clinical study | 104 | RR |
|  |  |  | CTR20160022 | Non-randomised, open, single-arm phase Ⅰ clinical study | 13 | RR |
| 92 | Axicabtagene Ciloleucel | Large B cell lymphoma | NCT03391466 | International multicentre, randomised, open, positive-controlled phase III clinical study | 359 | PFS |
| 93 | Orelabrutinib | Marginal zone lymphomas（MZL） | CTR20190011 | Non-randomised, open, single-arm, phase II clinical study | 83 | RR |
| 94 | Zimberelimab | Cervical Cancer | NCT03972722 | International multicentre, non-randomised, open, single-arm phase II clinical study | 90 | RR |
| 95 | Sugemalimab | Extranodal NK-T-Cell Lymphoma | CTR20180519 | Non-randomised, open, single-arm, phase II clinical study | 80 | RR |

Abbreviations: RR: Response Rate; OS: Overall Survival; PFS: Progression-Free Survival; DSN: Duration of severe neutropenia; tpCR: Total Physiological complex response.

## Supplementary Table S5. The characteristics of postmarketing studies.

| **Number** | **Drug Name** | **Trial Number** | **Trial Design** | Study Endpoints | Enrollment, No. | **Commencement of confirmatory clinical trials** | **Time Limits** | **Study Status** |
| --- | --- | --- | --- | --- | --- | --- | --- | --- |
| 1 | Zanubrutinib | CTR20201544 | International multicentre, randomised, open, positive drug-controlled phase III clinical study | PFS | 510 | 7/28/2019 | Yes  (3 years) | Completed |
| 2 | Ensartinib | NCT02767804 | International multicentre, randomised, open, positive drug-controlled phase III clinical study | PFS | 290 | 6/2020 | Yes (3 years) | Completed |
| 3 | Almonertinib | CTR20181951 | Randomised, double-blind, positive drug-controlled phase III clinical study | PFS | 429 | 11/30/2018 | None | Completed |
| 4 | Orelabrutinib | CTR20201980 | Randomised, open, positive drug-controlled phase III clinical study | PFS | 192 | 1/8/2021 | Yes  (3 years) | Running |
| 5 | Orelabrutinib | CTR20212348 | Randomised, open, positive drug-controlled phase III clinical study | PFS | 356 | 1/6/2022 | Yes  (3 years) | Running |
| 6 | Camrelizumab | CTR20200774 | Randomised, open, positive drug-controlled phase III clinical study | PFS | 56 | 7/16/2020 | Yes (5 years) | Running |
| 7 | Pralatrexate | NCT06072131 | International multicentre, randomised, open, positive drug-controlled phase III clinical study | PFS | 504 | 10/04/2023 | None | Running |
| 8 | Venetoclax | CTR20171498 | International multicentre, randomised, double-blind, placebo-controlled phase III clinical study | OS | 490 | 6/14/2018 | Yes (3 years) | Completed |
| 9 | Azvudine | CTR20221507 | Randomised, double-blind, placebo-controlled phase III clinical study | RNA <50copies/ml % of subjects | 720 | 6/8/2022 | Yes (5 years) | Running |
| 10 | Orebatinib | CTR20191420 | Randomised, open, positive drug-controlled phase II clinical study | PFS | 144 | 11/13/2019 | Yes (2 years) | Completed |
| 11 | Daratumumab | CTR20182174 | International multicentre, randomised, open, positive-controlled phase III clinical study | OS | 393 | 4/20/2019 | None | Running |
| 12 | Envafolimab | CTR20181127 | Non-randomised, open, single-arm phase II clinical study | RR | 103 | 8/3/2018 | Yes (5 years) | Running |
| 13 | Herombopag Olamine | CTR20190158 | Randomised, double-blind, placebo-controlled phase III clinical study | HR (⩾1 lineage; 18-week) | 120 | 4/4/2019 | None | Running |
| 14 | Furmonertinib | CTR20182519 | Randomised, double-blind, positive drug-controlled phase III clinical study | PFS | 358 | 5/31/2019 | Yes (5 years) | Completed |
| 15 | Pamiparib | CTR20171666 | Randomised, double-blind, positive drug-controlled phase III clinical study | PFS | 224 | 5/14/2018 | Yes (5 years) | Running |
| 16 | Penpulimab | CTR20211254 | Randomised, open, positive drug-controlled phase III clinical study | PFS | 60 | 3/1/2022 | None | Completed |
| 17 | Pralsetinib | CTR20190767 | International multicentre, non-randomised,open,single-arm Phase I/II clinical study | RR | 698 | 8/12/2019 | None | Completed |
| 18 | Relmacabtagene Autoleucel | CTR20220683 | Non-randomised, open, single-arm phase IV clinical study | RR | 41 | 1/13/2023 | Yes (3 years) | Running |
| 19 | Selinexor | CTR20211431 | Randomised, open, positive drug-controlled phase III clinical study | PFS | 154 | 7/27/2021 | None | Running |
| 20 | Savolitinib | NCT04923945 | International multicentre, non-randomised, open, single-arm phase III clinical study | RR | 203 | 8/19/2021 | Yes (5 years) | Running |
| 21 | Toripalimab | CTR20212889 | International multicentre, randomised, double-blind, placebo-controlled phase III clinical study | PFS | 559 | 11/1/2018 | Yes (5 years) | Completed |
| 22 | Tislelizumab | CTR20170882 | International multicentre, randomised, open, positive drug-controlled phase III clinical study | OS | 1085 | 3/1/2018 | Yes (5 years) | Completed |
| 23 | Disitamab Vedotin | CTR20202569 | Randomised, open, positive drug-controlled phase III clinical study | OS | 351 | 3/24/2021 | Yes (5 years) | Running |
| 24 | Disitamab Vedotin | CTR20220348 | Randomised, open, positive drug-controlled phase III clinical study | PFS | 452 | 6/8/2022 | Yes (5 years) | Running |
| 25 | Tislelizumab | CTR20180867 | Open, non-randomised ,single-arm phase Ⅱ study | RR | 200 | 9/19/2018 | Yes (5 years) | Running |
| 26 | Pembrolizumab | CTR20220264 | Randomised, open, positive drug-controlled phase III clinical study | PFS | 100 | 6/21/2022 | Yes (5 years) | Running |
| 27 | Tislelizumab | CTR20190543 | Randomised, double-blind, placebo-controlled phase III clinical study | OS | 332 | 5/29/2019 | Yes (5 years) | Running |
| 28 | Atezolizumab | CTR20213006 | Non-randomised, open, single-arm phase III clinical study | OS | 60 | 6/14/2022 | Yes (5 years) | Running |
| 29 | Olaparib | CTR20221020 | Randomised, open, positive drug-controlled phase Ⅳ clinical study | PFS | 43 | 12/30/2022 | Yes (5 years) | Running |
| 30 | Burosumab | CTR20210505 | Non-randomised, open, single-arm phase Ⅳ clinical study | Radiographic Global Impression of Change (RGI-C) Global Score at Week 40 | 28 | 11/1/2021 | Yes (5 years) | Running |
| 31 | Burosumab | CTR20221268 | Non-randomised, open, single-arm phase Ⅳ clinical study | Radiographic Global Impression of Change (RGI-C) Global Score at Week 40 | 9 | 9/7/2022 | Yes (5 years) | Running |
| 32 | Darolutamide | CTR20212232 | Non-randomised, open, single-arm phaseⅡ clinical study | RR | 60 | 1/13/2022 | Yes (5 years) | Running |
| 33 | Dinutuximab beta | CTR20220833 | Non-randomised, open, single-arm phase Ⅳ clinical study | PFS | 151 | 6/3/2022 | Yes (5 years) | Running |
| 34 | Gilteritinib | CTR20170326 | International multicentre, randomised, open, positive drug-controlled phase III clinical study | OS | 273 | 4/3/2018 | Yes (5 years) | Running |
| 35 | Nivolumab | CTR20212323 | Randomised, open, positive drug-controlled phaseⅡ clinical study | OS | 102 | 3/2/2022 | Yes (5 years) | Running |
| 36 | Mitoxantrone liposomal | CTR20202403 | Randomised, open, positive drug-controlled phase III clinical study | PFS | 190 | 5/13/2021 | None | Running |
| 37 | Ipilimumab | CTR20212323 | Randomised, open, positive drug-controlled phase Ⅱ clinical study | OS | 102 | 3/2/2022 | Yes (5 years) | Running |
| 38 | Icaritin | CTR20221588 | Randomised, double-blind, placebo-controlled phase III clinical study | OS | 261 | 3/28/2023 | Yes (3 years) | Running |
| 39 | Zanubrutinib | NCT03053440 | International multicentre, randomised, open， positive drug-controlled phase III clinical study | RR | 102 | 1/25/2017 | Yes (2 years) | Completed |
| 40 | Pyrotinib | CTR20191261 | Randomised, double-blind, placebo-controlled phase III clinical study | PFS | 2497 | 6/26/2019 | Yes (4 years) | Running |
| 41 | Serplulimab | CTR20190719 | Non-randomised, open, single-arm phaseⅡ clinical study | RR | 108 | 8/20/2019 | None | Running |
| 42 | Pucotenlimab | CTR20222431 | Randomised, open, placebo-controlled phase III clinical study | PFS | 190 | 5/30/2023 | Yes (4 years) | Running |
| 43 | Cadonilimab | CTR20211380 | Randomised, double-blind, placebo-controlled phase III clinical study | PFS | 440 | 9/11/2021 | Yes (4 years) | Running |
| 44 | Pucotenlimab | CTR20222648 | Randomised, open， positive drug-controlled phase III clinical study | PFS | 350 | 2/15/2023 | Yes (4 years) | Running |
| 45 | Pralsetinib | NCT04222972 | International multicentre, randomised, open，positive drug-controlled phase III clinical study | PFS | 221 | 7/24/2020 | None | Running |
| 46 | Larotrectinib | CTR20190625 | International multicentre, randomised, open, parallel-group phase Ⅱ clinical trial | RR | 259 | 5/12/2020 | Yes (6 years) | Running |
| 47 | Larotrectinib | CTR20190626 | International multicentre, randomised, open, single-arm phase Ⅰ/Ⅱ clinical trial | RR | 166 | 4/14/2020 | Yes (6 years) | Running |
| 48 | Mobocertinib | CTR20200164 | International multicentre, randomised, open，positive drug-controlled phase III clinical study | PFS | 430 | 6/22/2020 | None | Running |
| 49 | Pemigatinib | CTR20201757 | International multicentre, randomised, open，positive drug-controlled phase III clinical study | PFS | 65 | 5/24/2021 | Yes (5 years) | Running |
| 50 | Ivosidenib | CTR20181920 | International multicentre, randomised, double-blind, placebo-controlled phase III clinical study | PFS | 230 | 5/14/2019 | None | Completed |
| 51 | Trametinib | CTR20200259 | Randomised, open, single-arm phaseⅡ clinical study | RR | 40 | 8/29/2020 | Yes (4 years) | Running |
| 52 | Dabrafenib | CTR20200259 | Randomised, open, single-arm phaseⅡ clinical study | RR | 40 | 8/29/2020 | Yes (4 years) | Running |
| 53 | Entrectinib | CTR20201930 | International multicentre, non- randomised, open, single-arm phase Ⅱ clinical study | RR | 80 | 4/14/2021 | None | Running |
| 54 | Selpercatinib | CTR20192718 | International multicentre, randomised, open，positive drug-controlled phase III clinical study | PFS | 460 | 2/8/2021 | None | Running |
| 55 | Selpercatinib | CTR20192731 | International multicentre, randomised, open，positive drug-controlled phase III clinical study | PFS | 90 | 12/25/2020 | None | Running |
| 56 | Emapalumab | CTR20222253 | Non-randomised, open, single-arm phase Ⅳ clinical study | Permanent Discontinuation of Investigational Drug Due to Adverse Events Associated with Emapalumab | 18 | 1/29/2023 | None | Running |
| 57 | Luspatercept | CTR20222419 | Randomised, double-blind, parallel-group phase Ⅱ clinical study | Percentage of Participants Who Achieved Erythroid Response | 90 | 1/9/2023 | None | Running |
| 58 | Trodelvy | CTR20233370 | International multicentre, randomised, open，positive drug-controlled phase III clinical study | PFS | 757 | 11/23/2023 | Yes (4 years) | Completed |
| 59 | Mogamulizumab | CTR20230672 | Non-randomised, open, single-arm phase Ⅳ clinical study | RR | 20 | 5/29/2023 | None | Running |
| 60 | Polatuzumab Vedotin | CTR20181396 | International multicentre, randomised, double-blind，placebo-controlled, phase III clinical study | PFS | 1150 | 1/7/2019 | None | Completed |
| 61 | Glumetinib | CTR20231712 | Non-randomised, open, single-arm phase Ⅲ clinical study | RR | 161 | 10/11/2023 | None | Running |
| 62 | Camrelizumab | NCT03707509 | International multicentre, randomised, double-blind, placebo-controlled phase III clinical study | PFS | 263 | 11/13/2018 | None | Completed |
| 63 | Orelabrutinib | CTR20233094 | Randomised, double-blind, placebo-controlled phase III clinical study | PFS | 324 | 12/19/2023 | None | Running |
| 64 | Copanlisib | CTR20160362 | International multicentre, randomised, double-blind, placebo-controlled phase III clinical study | PFS | 649 | 8/1/2017 | None | Running |
| 65 | Acalabrutinib | NCT02972840 | International multicentre, randomised, double-blind, placebo-controlled phase III clinical study | PFS | 635 | 4/5/2017 | None | Running |
| 66 | Trilaciclib | CTR20210562 | Randomised, double-blind, placebo-controlled phase III clinical study | DSN | 95 | 5/25/2021 | None | Completed |
| 67 | Rezvilutamide | CTR20180560 | Randomised, open，positive drug-controlled phase III clinical study | PFS | 591 | 5/16/2021 | Yes (1 years) | Completed |
| 68 | Fluzoparib | CTR20190294 | Randomised, double-blind, placebo-controlled phase III clinical study | PFS | 252 | 4/30/2019 | Yes (3 years) | Completed |
| 69 | Zanubrutinib | CTR20190416 | International multicentre, randomised, open，positive drug-controlled phase III clinical study | PFS | 699 | 6/24/2019 | Yes (3 years) | Completed |
| 70 | Telitacicept | CTR20191388 | Randomised, double-blind, placebo-controlled phase III clinical study | Week 52 SLE Response Index 4 (SRI4) response rate | 335 | 10/16/2019 | None | Completed |
| 71 | Ripretinib | CTR20192728 | Non-randomised, open, single-arm phase Ⅱ clinical study | PFS | 39 | 4/23/2020 | Yes (1 years) | Completed |
| 72 | Blinatumomab | CTR20170176 | Non-randomised, open, single-arm phase III clinical study | RR | 121 | 10/18/2017 | Yes (5 years) | Completed |
| 73 | Avapritinib | CTR20191409 | Non-randomised, open, single-arm phase I/II clinical study | RR | 65 | 9/3/2019 | Yes (1 years) | Completed |
| 74 | Plerixafor | CTR20200882 | Non-randomised, open, single-arm phase Ⅳ clinical study | Proportion of patients with ≥6×10^6^ CD34+ cells/kg collected from peripheral blood on 2 or fewer collection days | 53 | 11/04/2021 | Yes (5 years) | Completed |
| 75 | Naxitamab | NCT03363373 | International multicentre, open, non-randomised,single-arm phase II clinical study | RR | 122 | 4/3/2018 | None | Running |
| 76 | Sunvozertinib | CTR20223235 | International multicentre, randomised, open，positive drug-controlled phase III clinical study | PFS | 512 | 12/13/2022 | None | Running |
| 77 | Vebreltinib | CTR20232228 | Non-randomised, open, single-arm phase III clinical study | RR | 131 | 9/30/2022 | None | Running |
| 78 | Axicabtagene | FKC876-2022-001 | Non-randomised,open, single-arm,phase Ⅱ clinical study | RR | / | / | Yes(4 years) | Running |
| 79 | Socazolimab | ZKAB001-LEES-2023-01 | Randomised, double-blind, placebo-controlled phase III clinical study | PFS | / | / | None | Running |
| 80 | Orelabrutinib | CTR20233094 | Randomised, double-blind, placebo-controlled phase III clinical study | PFS | 324 | 12/29/2023 | None | Running |
| 81 | Zimberelimab | GLS-010-302 | International multicentre, randomised, double-blind, placebo-controlled phase III clinical study | OS | / | / | None | Running |
| 82 | Sugemalimab | CS1001-306 | International multicentre, randomised, double-blind, placebo-controlled phase III clinical study | PFS | / | / | None | Running |
| 83 | Toripalimab | CTR20201856 | Randomised, double-blind, placebo-controlled phase III clinical study | PFS | 364 | 9/4/2020 | None | Running |
| 84 | Carfilzomib | CTR20190717 | Randomised, open，positive drug-controlled phase III clinical study | PFS | 123 | 11/28/2019 | None | Completed |

Abbreviations: Abbreviations: RR: Response Rate; OS: Overall Survival; PFS: Progression-Free Survival; DSN: Duration of severe neutropenia.

## Supplementary Table S6.The characteristics of drugs switched to regular approval.

| **Number** | **Drug Name** | **Conditional Approval Date** | **Time to start the confirmatory trial** | **Time to end the confirmatory trial** | **Supplementary application** | **Approved supplement** | **Time to convert to regular approval（days）** |
| --- | --- | --- | --- | --- | --- | --- | --- |
| 1 | Zanubrutinib | 6/2/2020 | 6/24/2019 | 5/7/2021 | 1/28/2022 | 4/28/2023 | 1060 |
| 2 | Blinatumomab | 12/2/2020 | 10/18/2017 | 6/7/2021 | 2/26/2021 | 4/13/2022 | 497 |
| 3 | Avapritinib | 3/30/2021 | 9/3/2019 | 4/11/2022 | 4/11/2022 | 2/3/2023 | 675 |
| 4 | Fluzoparib | 12/11/2020 | 4/30/2019 | 5/19/2022 | 9/8/2022 | 1/11/2023 | 791 |
| 5 | Camrelizumab | 4/27/2021 | 11/13/2018 | 6/15/2020 | 11/12/2020 | 6/8/2021 | 42 |
| 6 | Telitacicept | 3/9/2021 | 10/16/2019 | 4/24/2022 | 11/9/2021 | 4/24/2022 | 411 |
| 7 | Trilaciclib Hydrochloride | 7/12/2022 | 5/25/2021 | 1/1/2023 | 2/17/2023 | 10/27/2023 | 472 |
| 8 | Rezvilutamide | 6/28/2022 | 6/28/2018 | 5/16/2021 | 8/4/2022 | 3/22/2023 | 267 |
| 9 | Furmonertinib | 3/2/2021 | 5/31/2019 | 9/15/2021 | 12/17/2021 | 6/30/2022 | 485 |
| 10 | Ripretinib | 3/30/2021 | 4/23/2020 | 8/23/2022 | 11/24/2021 | 9/20/2022 | 539 |
| 11 | Plerixafor | 8/26/2020 | 11/4/2021 | 12/20/2022 | 4/11/2022 | 5/23/2023 | 1000 |
| 12 | Zanubrutinib | 6/2/2020 | 6/24/2019 | 5/7/2021 | 1/28/2022 | 4/28/2023 | 1060 |
| 13 | Zanubrutinib | 6/16/2021 | 1/25/2017 | 6/21/2022 | 1/28/2022 | 4/28/2023 | 654 |
| 14 | Ensartinib | 11/19/2020 | 6/30/2020 | / | 7/13/2021 | 3/22/2022 | 488 |
| 15 | Venetoclax | 12/2/2020 | 6/14/2018 | / | 1/11/2023 | 9/5/2023 | 1007 |
| 16 | Orebatinib | 11/26/2021 | 11/13/2019 | / | 7/19/2022 | 11/17/2023 | 721 |
| 17 | Pembrolizumab | 9/5/2023 | 8/13/2020 | / | 5/15/2024 | 7/5/2024 | 304 |
| 18 | Pralsetinib | 3/23/2021 | 8/12/2019 | / | 10/22/2022 | 6/27/2023 | 826 |
| 19 | Toripalimab | 2/10/2021 | 11/1/2018 | 11/18/2022 | 6/17/2023 | 8/21/2023 | 922 |
| 20 | Tislelizumab | 6/22/2021 | 3/1/2018 | 9/4/2023 | 12/27/2022 | 12/26/2023 | 917 |
| 21 | Carfilzomib | 7/6/2021 | 11/28/2019 | / | 11/25/2021 | 2/14/2023 | 588 |
| 22 | Trodelvy | 6/7/2022 | 11/23/2023 | / | 8/26/2024 | 11/6/2024 | 887 |
| 23 | Polatuzumab Vedotin | 1/10/2023 | 1/7/2019 | 2/7/2022 | 3/27/2024 | 6/3/2024 | 510 |
| 24 | Ivosidenib | 1/30/2022 | 5/14/2019 | / | 12/20/2023 | 8/26/2024 | 939 |
| 25 | Almonertinib | 3/17/2020 | 11/30/2018 | / | 10/9/2021 | 2/15/2022 | 700 |

## Supplementary Table S7. Drugs granted with application by China’s NMPA(2020-2023).

| **Number** | **Drug Name** | **Origin** | **Approval Path** | **Date to Clinical Approval** | **Application Date** | **Approval** Date | **Initial Approval Agency** | **Initial Approval Date** |
| --- | --- | --- | --- | --- | --- | --- | --- | --- |
| 1 | Blinatumomab | Imported | PR+CA | 9/17/2015 | 10/29/2019 | 12/2/2020 | FDA | 12/3/2014 |
| 2 | Ensartinib | Domestic | PR+CA | 10/1/2015 | 1/2/2019 | 11/17/2020 | NMPA | 11/17/2020 |
| 3 | Enzalutamide | Imported | PR | 3/13/2017 | 12/7/2019 | 11/2/2020 | FDA | 8/31/2012 |
| 4 | Abemaciclib | Imported | PR | 7/15/2014 | 11/14/2019 | 12/29/2020 | FDA | 9/28/2017 |
| 5 | Surufatinib | Domestic | PR | 6/9/2009 | 11/13/2019 | 12/29/2020 | NMPA | 12/29/2020 |
| 6 | Radium Ra 223 Dichloride | Imported | PR | 5/26/2012 | 8/23/2019 | 8/26/2020 | FDA | 5/15/2013 |
| 7 | Bevacizumab | Imported | RA | 9/16/2004 | 5/15/2019 | 9/14/2020 | FDA | 5/5/2009 |
| 8 | Trastuzumab Emtansine | Imported | PR | 7/2009 | 3/27/2019 | 1/21/2020 | FDA | 2/22/2013 |
| 9 | Atezolizumab | Imported | RA | 12/17/2013 | 2/25/2019 | 2/11/2020 | FDA | 5/18/2016 |
| 10 | Neratinib | Imported | RA | 8/17/2006 | 9/29/2018 | 4/27/2020 | FDA | 7/17/2017 |
| 11 | Plerixafor | Imported | PR+CA | / | 3/6/2020 | 8/26/2020 | FDA | 12/25/2008 |
| 12 | Orelabrutinib | Domestic | PR+CA | 6/21/2017 | 11/22/2019 | 12/29/2020 | NMPA | 12/29/2020 |
| 13 | Orelabrutinib | Domestic | PR+CA | 6/21/2017 | 11/22/2019 | 12/25/2020 | NMPA | 2/25/2020 |
| 14 | Venetoclax | Imported | PR+CA | 12/11/2015 | 1/13/2020 | 12/11/2020 | FDA | 4/11/2016 |
| 15 | Fuzuloparib | Domestic | PR+CA | 1/26/2019 | 10/29/2019 | 6/24/2020 | NMPA | 6/24/2020 |
| 16 | Brentuximab Vedotin | Imported | PR | 5/20/2013 | 4/28/2019 | 5/12/2020 | FDA | 8/19/2011 |
| 17 | Almonertinib | Domestic | PR+CA | 8/8/2016 | 4/18/2019 | 3/17/2020 | NMPA | 3/17/2020 |
| 18 | Pralatrexate | Imported | PR+CA | 9/10/2015 | 1/2/2019 | 8/26/2020 | FDA | 9/24/2009 |
| 19 | Zanubrutinib | Domestic | PR+CA | 2/3/2015 | 8/29/2018 | 6/2/2020 | FDA | 11/14/2019 |
| 20 | Zanubrutinib | Domestic | PR+CA | 2/3/2015 | 10/24/2018 | 6/2/2020 | FDA | 11/14/2019 |
| 21 | Camrelizumab | Domestic | PR+CA | 1/19/2015 | 5/16/2019 | 3/3/2020 | NMPA | 3/3/2020 |
| 22 | Tislelizumab | Domestic | PR+CA | 11/11/2015 | 6/3/2019 | 4/13/2020 | NMPA | 4/13/2020 |
| 23 | Pembrolizumab | Imported | PR | 5/6/2014 | 5/1/2020 | 12/8/2020 | FDA | 6/1/2019 |
| 24 | Niraparib | Domestic | PR | 3/1/2017 | 3/20/2020 | 9/8/2020 | FDA | 3/27/2017 |
| 25 | Apatinib | Domestic | RA | 4/1/2014 | 2/19/2020 | 12/29/2020 | NMPA | 12/29/2020 |
| 26 | Atezolizumab | Imported | PR | 5/3/2017 | 2/13/2020 | 10/27/2020 | EMA | 9/20/2017 |
| 27 | Lenvatinib | Imported | RA | 2/3/2015 | 12/7/2019 | 11/9/2020 | FDA | 2/13/2015 |
| 28 | Apalutamide | Imported | PR | 8/31/2016 | 12/5/2018 | 8/12/2020 | FDA | 2/14/2018 |
| 29 | Camrelizumab | Domestic | PR | 1/19/2015 | 9/12/2018 | 6/17/2020 | NMPA | 6/17/2020 |
| 30 | Nivolumab | Imported | PR | / | 7/3/2019 | 3/12/2020 | EMA | 6/19/2015 |
| 31 | Cetuximab | Imported | PR | / | 4/15/2019 | 2/26/2020 | EMA | 11/1/2008 |
| 32 | Ceritinib | Imported | RA | 12/8/2014 | 1/14/2019 | 5/26/2020 | FDA | 4/29/2014 |
| 33 | Disitamab Vedotin | Domestic | PR+CA | 12/31/2015 | 8/28/2020 | 6/8/2021 | NMPA | 6/8/2021 |
| 34 | Selinexor | Imported | PR+CA | 11/26/2018 | 1/28/2021 | 12/24/2021 | FDA | 7/3/2019 |
| 35 | Envafolimab | Domestic | PR+CA | 3/31/2017 | 12/21/2020 | 11/24/2021 | NMPA | 11/24/2021 |
| 36 | Dalpiciclib | Domestic | PR+BTD | 6/16/2014 | 4/27/2021 | 12/31/2021 | NMPA | 12/31/2021 |
| 37 | Sonidegib | Imported | PR | / | 2/19/2021 | 7/20/2021 | FDA | 7/24/2015 |
| 38 | Pembrolizumab | Imported | PR+CA | / | 12/14/2020 | 6/8/2021 | FDA | 9/4/2014 |
| 39 | Nivolumab | Imported | PR+CA | 9/7/2017 | 12/3/2020 | 6/8/2021 | EMA | 6/19/2015 |
| 40 | Ipilimumab | Imported | PR+CA | 5/25/2017 | 12/3/2020 | 6/8/2021 | FDA | 3/25/2011 |
| 41 | Sugemalimab | Domestic | RA | 9/6/2017 | 11/13/2020 | 12/20/2021 | NMPA | 12/20/2021 |
| 42 | Dinutuximab | Imported | PR+CA | / | 11/4/2020 | 8/12/2021 | EMA | 5/8/2017 |
| 43 | Donafenib | Domestic | PR | 12/28/2011 | 5/15/2020 | 6/8/2021 | NMPA | 6/8/2021 |
| 44 | Avapritinib | Imported | PR+CA | 11/26/2018 | 4/29/2020 | 3/30/2021 | FDA | 1/9/2020 |
| 45 | Darolutamide | Imported | PR+CA | / | 2/15/2020 | 2/2/2021 | FDA | 7/30/2019 |
| 46 | Inotuzumab ozogamicin | Imported | PR | / | 1/22/2020 | 12/20/2021 | EMA | 6/28/2017 |
| 47 | Obinutuzumab | Imported | PR | 3/23/2010 | 9/28/2019 | 6/1/2021 | FDA | 11/1/2013 |
| 48 | Utidelone | Domestic | PR | 4/28/2006 | 3/28/2018 | 3/11/2021 | NMPA | 3/11/2021 |
| 49 | Olverembatinib | Domestic | PR+CA | 5/15/2015 | 10/10/2020 | 11/24/2021 | NMPA | 11/24/2021 |
| 50 | Pralsetinib | Imported | PR+CA | 1/9/2019 | 9/8/2020 | 3/23/2021 | FDA | 9/4/2020 |
| 51 | Ripretinib | Imported | PR+CA | 9/18/2019 | 7/22/2020 | 3/30/2021 | FDA | 5/15/2020 |
| 52 | Pamiparib | Domestic | PR+CA | 11/4/2015 | 7/20/2020 | 4/30/2021 | NMPA | 4/30/2021 |
| 53 | Relmacabtagene Autoleucel | Domestic | PR+CA | 2/28/2018 | 6/30/2020 | 9/1/2021 | NMPA | 9/1/2021 |
| 54 | Savolitinib | Domestic | PR+CA | 4/14/2015 | 6/6/2020 | 6/22/2021 | NMPA | 6/22/2021 |
| 55 | Penpulimab | Domestic | CA | 8/21/2017 | 5/28/2020 | 8/3/2021 | NMPA | 8/3/2021 |
| 56 | Axicabtagene Ciloleucel | Domestic | PR | 5/1/2018 | 2/26/2020 | 6/22/2021 | NMPA | 6/22/2021 |
| 57 | Zimberelimab | Domestic | CA | 3/15/2017 | 2/21/2020 | 8/25/2021 | NMPA | 8/25/2021 |
| 58 | Furmonertinib | Domestic | PR+CA | 9/3/2016 | 12/9/2019 | 3/2/2021 | NMPA | 3/2/2021 |
| 59 | Carfilzomib | Imported | CA | 3/31/2017 | 11/30/2019 | 7/6/2021 | FDA | 7/20/2012 |
| 60 | Geretinib | Imported | PR+CA | / | 4/11/2020 | 1/30/2021 | FDA | 11/28/2018 |
| 61 | Olaparib | Imported | PR+CA | / | 1/12/2021 | 6/16/2021 | EMA | 12/16/2014 |
| 62 | Atezolizumab | Imported | PR+CA | 8/24/2017 | 9/23/2020 | 4/29/2021 | FDA | 5/18/2016 |
| 63 | Tislelizumab | Domestic | CA | 11/11/2015 | 6/29/2020 | 6/22/2021 | NMPA | 6/22/2021 |
| 64 | Toripalimab | Domestic | PR+CA | 3/20/2018 | 5/7/2020 | 2/10/2021 | NMPA | 2/10/2021 |
| 65 | Toripalimab | Domestic | PR+CA | 1/14/2016 | 5/21/2020 | 4/7/2021 | NMPA | 4/7/2021 |
| 66 | Anlotinib | Domestic | PR+CA | 7/3/2015 | 12/13/2019 | 1/30/2021 | NMPA | 1/30/2021 |
| 67 | Zanubrutinib | Domestic | PR+CA | 2/13/2015 | 9/30/2020 | 6/16/2021 | FDA | 11/14/2019 |
| 68 | Daratumumab | Imported | PR+CA | 9/18/2016 | 11/6/2020 | 9/30/2021 | EMA | 5/20/2016 |
| 69 | Camrelizumab | Domestic | PR+CA | 8/4/2018 | 9/4/2020 | 4/27/2021 | NMPA | 4/27/2021 |
| 70 | Disitamab Vedotin | Domestic | BTD+PR+CA | 1/7/2019 | 7/14/2021 | 12/31/2021 | NMPA | 12/31/2021 |
| 71 | Almonertinib | Domestic | BTD+PR | 8/8/2016 | 5/20/2021 | 12/24/2021 | NMPA | 12/24/2021 |
| 72 | Bevacizumab | Imported | RA | / | 4/7/2021 | 11/17/2021 | FDA | 9/14/2017 |
| 73 | Bevacizumab | Domestic | PR | 4/21/2015 | 1/20/2021 | 6/25/2021 | FDA | 2/26/2004 |
| 74 | Bevacizumab | Domestic | RA | / | 6/24/2020 | 11/17/2021 | NMPA | 11/17/2021 |
| 75 | Camrelizumab | Domestic | RA | 1/19/2015 | 3/26/2021 | 12/8/2021 | NMPA | 12/8/2021 |
| 76 | Tislelizumab | Domestic | RA | 12/18/2016 | 3/5/2021 | 12/31/2021 | NMPA | 12/31/2021 |
| 77 | Tislelizumab | Domestic | RA | 12/18/2016 | 4/23/2020 | 1/12/2021 | NMPA | 1/12/2021 |
| 78 | Toripalimab | Domestic | RA | 12/27/2015 | 2/11/2021 | 11/24/2021 | NMPA | 11/24/2021 |
| 79 | Daratumumab | Imported | RA | 9/18/2016 | 2/19/2021 | 11/9/2021 | FDA | 11/16/2015 |
| 80 | Sintilimab | Domestic | PR | 1/25/2016 | 1/13/2021 | 6/25/2021 | NMPA | 6/25/2021 |
| 81 | Abemaciclib | Imported | RA | 3/19/2017 | 12/28/2020 | 12/31/2020 | FDA | 9/28/2017 |
| 82 | Nivolumab | Imported | RA | 5/20/2013 | 12/25/2020 | 8/25/2021 | EMA | 6/19/2015 |
| 83 | Fuzuloparib | Domestic | PR | 11/7/2018 | 12/24/2021 | 6/22/2021 | NMPA | 6/22/2021 |
| 84 | Pembrolizumab | Imported | RA | 5/6/2014 | 11/23/2020 | 9/1/2021 | FDA | 9/4/2021 |
| 85 | Camrelizumab | Domestic | PR | 1/19/2015 | 11/12/2020 | 6/8/2021 | NMPA | 6/8/2021 |
| 86 | Gemcitabine | Imported | RA | 7/5/2018 | 11/9/2020 | 7/20/2021 | FDA | 2/15/1996 |
| 87 | Surufatinib | Domestic | RA | 12/26/2021 | 9/19/2019 | 6/16/2021 | NMPA | 6/16/2021 |
| 88 | Sintilimab | Domestic | RA | 1/25/2016 | 8/20/2020 | 6/1/2021 | NMPA | 6/1/2021 |
| 89 | Sintilimab | Domestic | RA | 1/25/2016 | 4/26/2020 | 2/2/2021 | NMPA | 2/2/2021 |
| 90 | Durvalumab | Imported | RA | 2/11/2015 | 7/24/2020 | 7/12/2021 | FDA | 5/1/2017 |
| 91 | Pemetrexed Disodium | Imported | RA | 8/23/2011 | 6/15/2020 | 6/8/2021 | EMA | 9/14/2015 |
| 92 | Pemetrexed Disodium | Imported | RA | 8/23/2011 | 7/4/2020 | 6/8/2021 | EMA | 9/14/2015 |
| 93 | Brentuximab Vedotin | Imported | RA | / | 7/4/2020 | 4/13/2021 | FDA | 8/19/2021 |
| 94 | Trastuzumab Emtansine | Imported | RA | 2/24/2017 | 5/27/2020 | 6/22/2021 | FDA | 2/22/2013 |
| 95 | Lenalidomide | Imported | PR | 7/16/2013 | 12/2/2019 | 3/16/2021 | PMDA | 12/2015 |
| 96 | Levofolinic | Domestic | RA | 3/29/2010 | 4/16/2018 | 6/30/2021 | NMPA | 6/30/2021 |
| 97 | Trilaciclib | Imported | PR+CA | 11/18/2020 | 12/1/2021 | 7/12/2022 | FDA | 2/12/2021 |
| 98 | Selpercatinib | Imported | PR+CA | 9/26/2019 | 11/9/2021 | 9/30/2022 | FDA | 5/8/2020 |
| 99 | Selpercatinib | Imported | PR+CA | 9/26/2019 | 11/9/2021 | 9/30/2022 | FDA | 5/8/2020 |
| 100 | Pucotenlimab | Domestic | PR+CA | 8/28/2017 | 7/5/2021 | 9/20/2022 | NMPA | 9/20/2022 |
| 101 | Pucotenlimab | Domestic | PR+CA | 2/22/2017 | 10/26/2021 | 7/19/2022 | NMPA | 7/19/2022 |
| 102 | Entrectinib | Imported | PR+CA | 12/17/2018 | 10/29/2021 | 7/26/2022 | FDA | 8/15/2019 |
| 103 | Cadonilimab | Domestic | BTD+PR+CA | 6/8/2018 | 9/26/2021 | 6/28/2022 | NMPA | 6/28/2022 |
| 104 | Trametinib | Imported | PR+CA | 8/27/2019 | 9/6/2021 | 3/22/2022 | EMA | 6/30/2014 |
| 105 | Dabrafenib | Imported | PR+CA | 8/21/2019 | 9/6/2021 | 3/22/2022 | FDA | 5/29/2013 |
| 106 | Ivosidenib | Imported | PR+CA | 6/25/2018 | 8/3/2021 | 1/30/2022 | FDA | 7/20/2018 |
| 107 | Naxitamab | Imported | PR+CA | 12/6/2017 | 7/14/2021 | 11/30/2022 | FDA | 11/25/2020 |
| 108 | Pemigatinib | Imported | PR+CA | 8/23/2019 | 7/12/2021 | 3/29/2022 | FDA | 4/17/2020 |
| 109 | Sacituzumab | Imported | PR+CA | 1/22/2020 | 5/18/2021 | 6/7/2022 | FDA | 4/22/2020 |
| 110 | linperlisib | Domestic | PR+CA+BTD | 12/21/2016 | 5/7/2021 | 11/8/2022 | NMPA | 11/8/2022 |
| 111 | Serplulimab | Domestic | PR+CA | 3/6/2018 | 4/23/2021 | 3/22/2022 | NMPA | 3/22/2022 |
| 112 | Duvelisib | Imported | PR+CA | 5/9/2019 | 4/21/2021 | 3/16/2022 | FDA | 9/24/2018 |
| 113 | Emapalumab | Imported | PR+CA | 4/1/2020 | 12/10/2020 | 3/8/2022 | FDA | 11/20/2018 |
| 114 | Rezvilutamide | Domestic | PR+CA+BTD | 4/19/2016 | 11/1/2021 | 6/28/2022 | NMPA | 6/28/2022 |
| 115 | Lorlatinib | Imported | PR+CA | 3/13/2017 | 4/15/2015 | 4/27/2022 | FDA | 11/2/2018 |
| 116 | Mogamulizumab | Imported | PR+CA | / | 7/2/2021 | 10/11/2022 | FDA | 8/8/2018 |
| 117 | Larotrectinib | Imported | PR+CA | 1/14/2019 | 5/20/2021 | 4/8/2022 | FDA | 11/26/2018 |
| 118 | Larotrectinib | Imported | PR+CA | 1/14/2019 | 5/20/2021 | 6/23/2022 | FDA | 11/26/2018 |
| 119 | Trastuzumab | Imported | RA | / | 4/21/2021 | 9/30/2022 | FDA | 6/29/2020 |
| 120 | Icaritin | Domestic | PR+CA | 1/1/2013 | 4/10/2021 | 1/10/2022 | NMPA | 1/10/2022 |
| 121 | Brigatinib | Imported | RA | 8/22/2018 | 1/21/2021 | 3/22/2022 | FDA | 4/28/2017 |
| 122 | Ramucirumab | Imported | RA | 2/3/2010 | 1/21/2021 | 3/16/2022 | FDA | 4/21/2014 |
| 123 | Irinotecan | Imported | RA | / | 6/22/2020 | 4/12/2022 | PMDA | 6/26/2009 |
| 124 | Procarbazine | Imported | RA | 5/9/2011 | 5/11/2020 | 4/19/2022 | PMDA | 3/7/2005 |
| 125 | Ripertamab | Domestic | RA | 6/6/2016 | 12/16/2019 | 8/23/2022 | NMPA | 8/23/2022 |
| 126 | Pralsetinib | Imported | PR+CA+BTD | 1/9/2019 | 3/29/2021 | 3/8/2022 | FDA | 9/4/2020 |
| 127 | Blinatumomab | Imported | PR+CA | / | 3/31/2021 | 4/27/2022 | FDA | 12/3/2014 |
| 128 | Pyrotinib | Domestic | PR+CA | 5/6/2019 | 9/15/2021 | 5/31/2022 | NMPA | 5/31/2022 |
| 129 | Mitoxantrone | Domestic | PR+CA | 5/19/2014 | 8/27/2020 | 1/7/2022 | NMPA | 1/7/2022 |
| 130 | Tislelizumab | Domestic | PR+CA | 11/11/2015 | 6/4/2021 | 3/8/2022 | NMPA | 3/8/2022 |
| 131 | Relmacabtagene Autoleucel | Domestic | PR+CA+BTD | 1/12/2018 | 3/2/2022 | 9/30/2022 | NMPA | 9/30/2022 |
| 132 | Pembrolizumab | Imported | RA | / | 3/8/2022 | 11/1/2022 | FDA | 9/4/2014 |
| 133 | Furmonertinib | Domestic | PR+BTD | 1/25/2016 | 12/27/2021 | 6/28/2022 | NMPA | 6/28/2022 |
| 134 | Toripalimab | Domestic | RA | 12/27/2015 | 7/30/2021 | 5/10/2022 | NMPA | 5/10/2022 |
| 135 | Toripalimab | Domestic | RA | 12/27/2015 | 12/24/2021 | 9/14/2022 | NMPA | 9/14/2022 |
| 136 | Pembrolizumab | Imported | RA | 8/29/2016 | 11/26/2021 | 9/30/2022 | FDA | 9/4/2014 |
| 137 | Sintilimab | Domestic | RA | 1/25/2016 | 11/4/2021 | 6/23/2022 | NMPA | 6/23/2022 |
| 138 | Cetuximab | Imported | RA | 5/28/2015 | 11/3/2021 | 6/16/2022 | PMDA | 9/25/2020 |
| 139 | Donafenib | Domestic | RA | 12/18/2011 | 10/21/2021 | 8/10/2022 | NMPA | 8/10/2022 |
| 140 | Serplulimab | Domestic | RA | 9/16/2019 | 9/16/2021 | 10/25/2022 | NMPA | 10/25/2022 |
| 141 | Nivolumab | Imported | RA | 5/30/2016 | 9/16/2021 | 6/23/2022 | EMA | 6/19/2015 |
| 142 | Sugemalimab | Domestic | RA | 6/1/2018 | 9/6/2021 | 5/31/2022 | NMPA | 5/31/2022 |
| 143 | Ramucirumab | Imported | RA | 6/21/2017 | 8/23/2021 | 9/30/2022 | EMA | 12/19/2014 |
| 144 | Tislelizumab | Domestic | RA | 12/18/2016 | 8/20/2021 | 6/7/2022 | NMPA | 6/7/2022 |
| 145 | Tislelizumab | Domestic | RA | 12/18/2016 | 7/8/2021 | 4/8/2022 | NMPA | 4/8/2022 |
| 146 | Atezolizumab | Imported | RA | 2/16/2016 | 6/30/2021 | 3/16/2022 | FDA | 10/18/2016 |
| 147 | Anlotinib | Domestic | RA | 7/1/2010 | 5/7/2021 | 4/8/2022 | NMPA | 4/8/2022 |
| 148 | Everolimus | Imported | RA | 2/13/2015 | 4/23/2020 | 1/30/2022 | FDA | 3/30/2009 |
| 149 | Telpegfilgrastim | Domestic | RA | 3/1/2010 | 4/29/2022 | 6/30/2023 | NMPA | 6/30/2023 |
| 150 | Adebrelimab | Domestic | RA | 2/20/2017 | 1/19/2022 | 2/28/2023 | NMPA | 2/28/2023 |
| 151 | Zuberitamab | Domestic | RA | 1/26/2015 | 1/11/2022 | 5/12/2023 | NMPA | 5/12/2023 |
| 152 | Efbemalenograstim | Domestic | RA | 1/1/2011 | 2/25/2022 | 5/6/2023 | NMPA | 5/6/2023 |
| 153 | Polatuzumab | Imported | PR+CA | 2/2/2018 | 12/30/2021 | 1/10/2023 | FDA | 6/10/2019 |
| 154 | Acalabrutinib | Imported | CA | 6/4/2018 | 1/5/2022 | 3/21/2023 | FDA | 10/31/2017 |
| 155 | Glumetinib | Domestic | BTD+PR+CA | 9/8/2020 | 2/5/2022 | 3/7/2023 | NMPA | 3/7/2023 |
| 156 | Mobocertinib | Imported | BTD+PR+CA | 12/24/2018 | 7/12/2021 | 1/10/2023 | FDA | 9/15/2021 |
| 157 | Copanlisib | Imported | PR+CA | 8/28/2015 | 3/10/2021 | 5/19/2023 | FDA | 9/14/2017 |
| 158 | Befotertinib | Domestic | RA | 5/24/2018 | 3/4/2021 | 5/29/2023 | NMPA | 5/29/2023 |
| 159 | Ribociclib | Imported | RA | 8/15/2018 | 10/22/2021 | 1/19/2023 | FDA | 3/13/2017 |
| 160 | Glofitamab | Imported | BTD+PR+CA | 7/3/2020 | 2/1/2023 | 11/7/2023 | FDA | 6/15/2023 |
| 161 | Vebreltinib | Domestic | BTD+PR+CA | 4/27/2016 | 9/24/2022 | 11/17/2023 | NMPA | 11/17/2023 |
| 162 | Equecabtagene Autoleucel | Domestic | BTD+PR+CA | 6/21/2019 | 6/6/2022 | 6/30/2023 | NMPA | 6/30/2023 |
| 163 | Inaticabtagene Autoleucel | Domestic | BTD+PR+CA | 3/4/2020 | 12/13/2022 | 11/7/2023 | NMPA | 11/7/2023 |
| 164 | Narlumosbart | Domestic | PR+CA | 8/11/2016 | 6/22/2022 | 9/5/2023 | NMPA | 9/5/2023 |
| 165 | Socazolimab | Domestic | BTD+CA | 5/17/2017 | 10/27/2021 | 12/19/2023 | NMPA | 12/19/2023 |
| 166 | Sunvozertinib | Domestic | BTD+PR+CA | 4/30/2019 | 1/10/2023 | 8/22/2023 | NMPA | 8/22/2023 |
| 167 | Zimberelimab | Domestic | CA | 6/6/2016 | 3/15/2022 | 6/30/2023 | NMPA | 6/30/2023 |
| 168 | Sugemalimab | Domestic | BTD+PR+CA | 6/1/2018 | 9/8/2022 | 10/27/2023 | NMPA | 10/27/2023 |
| 169 | Axicabtagene Ciloleucel | Domestic | PR+CA | 1/5/2018 | 10/25/2022 | 6/21/2023 | NMPA | 6/21/2023 |
| 170 | Ribociclib | Imported | RA | 2/25/2019 | 9/24/2021 | 5/19/2023 | FDA | 3/13/2017 |
| 171 | Trastuzumab Deruxtecan | Imported | BTD+PR | 11/21/2018 | 3/21/2022 | 2/21/2023 | FDA | 12/20/2019 |
| 172 | Camrelizumab | Domestic | RA | 1/19/2015 | 12/25/2021 | 1/29/2023 | NMPA | 1/29/2023 |
| 173 | Zanubrutinib | Domestic | RA | 10/23/2019 | 1/28/2022 | 4/28/2023 | FDA | 11/14/2019 |
| 174 | Selumetinib Hydrogen | Imported | PR | 11/20/2019 | 6/13/2022 | 4/28/2023 | FDA | 4/10/2022 |
| 175 | Daratumumab | Imported | RA | 6/6/2019 | 4/15/2021 | 5/19/2023 | FDA | 11/6/2015 |
| 176 | Aponermin | Domestic | RA | 1/21/2005 | 12/20/2021 | 11/1/2023 | NMPA | 11/1/2023 |
| 177 | Margetuximab | Imported | RA | 11/16/2019 | 1/7/2022 | 8/29/2023 | FDA | 12/16/2020 |
| 178 | Orelabrutinib | Domestic | PR+CA | 6/21/2017 | 8/15/2022 | 4/17/2023 | NMPA | 4/17/2023 |

Abbreviations: NMPA: National Medical Products Administration; FDA: Food and Drug Administration; EMA: European Medicines Agency; PMDA: Pharmaceuticals and Medical Devices Agency; CA: Conditional Approval; BTD: Breakthrough Therapy Designation; PR: Priority Review. “/”indicates the approvals based on data from overseas clinical trials.
